# The calcium channel subunit gamma-4 is regulated by MafA and necessary for pancreatic beta-cell specification

**DOI:** 10.1038/s42003-019-0351-4

**Published:** 2019-03-15

**Authors:** Cheng Luan, Yingying Ye, Tania Singh, Mohammad Barghouth, Lena Eliasson, Isabella Artner, Enming Zhang, Erik Renström

**Affiliations:** 1 Lund University Diabetes Center, Department of Clinical Sciences Malmö, Lund University, 202 13 Malmö, Sweden; 2 Stem Cell Center, Department of Laboratory Medicine, Lund University, 221 85 Lund, Sweden

## Abstract

Voltage-gated Ca^2+^ (Ca_V_) channels trigger glucose-induced insulin secretion in pancreatic beta-cell and their dysfunction increases diabetes risk. These heteromeric complexes include the main subunit alpha1, and the accessory ones, including subunit gamma that remains unexplored. Here, we demonstrate that Ca_V_ gamma subunit 4 (Ca_V_γ4) is downregulated in islets from human donors with diabetes, diabetic Goto-Kakizaki (GK) rats, as well as under conditions of gluco-/lipotoxic stress. Reduction of Ca_V_γ4 expression results in decreased expression of L-type Ca_V_1.2 and Ca_V_1.3, thereby suppressing voltage-gated Ca^2+^ entry and glucose stimulated insulin exocytosis. The most important finding is that Ca_V_γ4 expression is controlled by the transcription factor responsible for beta-cell specification, MafA, as verified by chromatin immunoprecipitation and experiments in beta-cell specific MafA knockout mice (*MafA*^*Δβcell*^). Taken together, these findings suggest that Ca_V_γ4 is necessary for maintaining a functional differentiated beta-cell phenotype. Treatment aiming at restoring Ca_V_γ4 may help to restore beta-cell function in diabetes.

## Abstract (web summary)

Cheng Luan et al. report that the voltage-gated calcium channel Ca_V_γ4 is necessary for maintaining pancreatic beta-cell function. They find that MafA, a transcription factor required for beta-cell maturation, directly regulates the gene encoding Ca_V_γ4 and suggest that restoration of Ca_V_γ4 may be a potential treatment for type 2 diabetes.

## Introduction

Pancreatic beta-cell failure is central in diabetes mellitus^1^. Deteriorated insulin secretion arises from a damaged secretory machinery or reduced beta-cell mass, collectively referred to as functional beta-cell mass. Insulin secretion is triggered by Ca^2+^ entry via voltage-gated Ca^2+^ (Ca_V_) channels, activation of the Ca^2+^ sensor synaptotagmin, and exocytosis of docked insulin granules^2^. Ca_V_ channels are heteromeric complexes including the pore-forming alpha1 subunits and accessory beta, alpha2delta and gamma subunits^3^. The best-studied are Ca_V_ alpha1 that have specific roles in phasic insulin secretion^3,4^. The accessory Ca_V_ beta and alpha2delta determine ion channel trafficking and membrane targeting^5,6^. However, the least studied are the eight Ca_V_ gamma (Ca_V_γ) variants, and reports are contradictory^7^. For example, gamma1 inhibits L-type Ca_V_1.1 channels in skeletal muscle, but physically associates with cardiac L-type Ca_V_1.2 (refs. ^8,9^). Notably, Ca_V_γ subunits were shown to bind with voltage-sensing domain IV (VSD_IV_) in alpha1 subunits^10^. The functions of the voltage-gated Ca^2+^ channel subunit gamma4 (Ca_V_γ4) are largely unknown, but it is widely detected in many tissues^11^.

Furthermore, Ca^2+^ signals influence functional beta-cell mass and development of diabetes. For instance, common genetic variations in the genes encoding L-type Ca_V_1.3 and R-type Ca_V_2.3 affect beta-cell functionality or risk of type-2 diabetes (T2D)^12,13^. Beta-cell mass reduction leading to diabetes may be induced by apoptosis as a consequence of, e.g., autoimmunity or endoplasmic reticulum (ER) stress^14^. However, recent evidence suggests that functional beta-cell mass reduction may be due to beta-cell dedifferentiation rather than cell death^15^. Previous reports point to Ca^2+^ signaling by Ca_V_ channels being a regulator of beta-cell mass through control of differentiation. For example, ablation of the alpha1 subunit Ca_V_1.3 in mice decreases both the number and size of islets compared to wild type, without affecting beta-cell death^16^. In Ca_V_2.3 knockout mice, islet cell differentiation also seemed imperfect, suggestive of a role in beta-cell differentiation^4^. Additionally, pharmacological inhibition of Ca_V_ channels in neonatal rat pancreata by diltiazem markedly decreases beta-cell proliferation^17^. Concerning the Ca_V_γ, gamma4 is the only one shown to be involved in differentiation, e.g., in the fetal brain and differentiating myoblasts^18^. Collectively, these reports show that Ca_V_ channels influence the signaling pathway of differentiation, but the underlying molecular mechanisms remain unknown.

Several transcription factors control pancreatic cell lineage differentiation and specification, e.g. Pdx1, necessary for pancreas development, Nkx6.1, and MafA that determine the final maturation into beta cells^19,20^. Genes targeted by MafA include Ca^2+^ signaling molecules, such as Ca^2+^/CamkII^20,21^. In line with the idea that beta-cell loss in T2D occurs by dedifferentiation rather than apoptosis, Pdx1, MafA, and Nkx6.1 are downregulated under such conditions^15,22,23^.

In this study, we have employed systems biology approaches to address the roles Ca_V_ channels play in beta-cell differentiation, in combination with physiological validation that identifies the Ca_V_γ4 as a downstream target of MafA. Ca_V_γ4 controls expression of other Ca_V_ subunits and affects Ca_V_ channel electrophysiology. Downregulation of Ca_V_γ4 occurs in islets from patients with T2D and from diabetic animal models, which suppresses insulin secretion. In conclusion, our results demonstrate that Ca_V_γ4 affects insulin exocytosis, but plays an even more important role in maintaining beta-cell differentiation and islet health.

## Results

### Ca_V_γ4 expression is reduced by gluco-/lipotoxic treatment

**Fig. 1:**
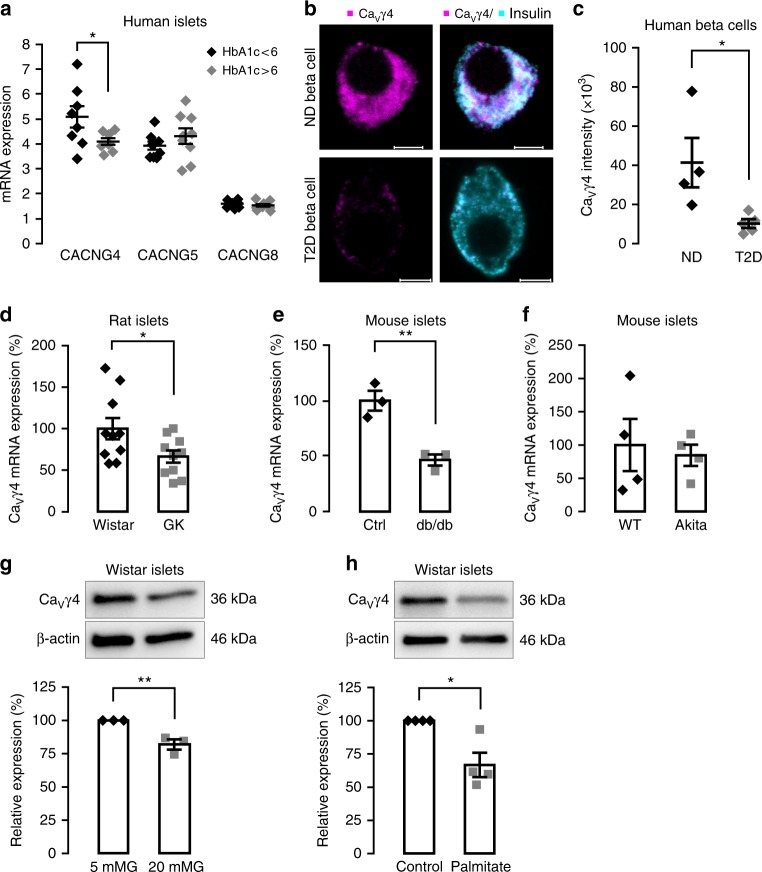
Decreased Ca_V_γ4 expression in beta cells in diabetes and in response to glucotoxicity. **a** Ca_V_γ4 (*CACNG4*), Ca_V_γ5 (*CACNG5*), and Ca_V_γ8 (*CACNG8*) mRNA expression in human islets from donors with HbA1c <6 and >6. n = 8 or 9 donors with age and gender matched, *p = 0.045 (*CACNG4*), *p* = 0.294 (*CACNG5*), *p* = 0.395 (*CACNG8*). **b** Representative immunofluorescence images of Ca_V_γ4 expression in human islet beta cells from non-diabetic (ND) and T2D donor. Scale: 5 μm. Ca_V_γ4 in magenta and insulin in cyan (7–11 beta cells were analyzed per each T2D donor, and 5–7 beta cells for each ND donor). **c** Calculation of fluorescent intensity for Ca_V_γ4. *n* = 4 ND and 5 T2D donors (5–11 beta cells were analyzed per donor), *p = 0.029. **d** Decreased Ca_V_γ4 mRNA expression in Goto-Kakizaki (GK) rat islets. *n* = 10 rats each, *p = 0.036. **e** As in **d** but in *db/db* mouse islets. *n* = 3 mice each, **p = 0.006. **f** Ca_V_γ4 mRNA expression in wild type and Akita mouse islets. *n* = 4 mice each, *p* = 0.727. **g** Decreased Ca_V_γ4 protein expression in Wistar rat islets cultured at 5 or 20 mM glucose (72 h). *n* = 3, **p = 0.009. **h** As in **g** but cultured with 1 mM palmitate (48 h). *n* = 4, *p = 0.011. Data are presented as mean ± SEM and were analyzed with two-tailed unpaired Student’s *t-*test. See also Supplementary Table 1 for the details of the human donors utilized for experiments in this study. WT wild type, Ctrl control

Analysis of human pancreatic islets mRNA microarray database^24^ displayed that among all Ca_V_γ subunits Ca_V_γ4 (*CACNG4*) was notably downregulated in individuals with higher glycated hemoglobin (HbA1c) values (Supplementary Fig. 1a). Altered Ca_V_γ4 expression was confirmed in human islets by qPCR, but not for Ca_V_γ5 nor Ca_V_γ8 (*p* = 0.045, 0.294, and 0.395, respectively; Fig. 1a. See also Supplementary Table 1 for the characteristics of human islet donors used experimentally in current study). Confocal immunocytochemistry revealed a marked loss of Ca_V_γ4 expression in beta cells from T2D human donors (*p* = 0.029; Fig. 1b, c). We then investigated several hyperglycemic or diabetic animal models. Interestingly, reduction of Ca_V_γ4 expression was found in islets from Goto-Kakizaki (GK) rats and *db/db* mice (*p* = 0.036 and 0.006, respectively; Fig. 1d, e), but not in insulin mutant Akita mice (Fig. 1f). To establish these in vivo findings, we next determined Ca_V_γ4 expression under gluco-/lipotoxic conditions in vitro. Seventy-two hours culture in 20 mM glucose or 48 h culture with 1 mM palmitate both decreased Ca_V_γ4 protein expression in healthy Wistar rat islets (*p* = 0.009 and 0.011, respectively; Fig. 1g, h). Accordingly, Ca_V_γ4 protein expression in human islets was also reduced after incubation in 20 mM glucose for 48 h (Supplementary Fig. 1b), but not for 72 h (Supplementary Fig. 7l), implying a compensatory mechanism for maintaining stable Ca_V_γ4 expression in human healthy beta cells under conditions of glucotoxicity. A similar reduction of Ca_V_γ4 was found in rat insulinoma INS-1 832/13 cells (Supplementary Fig. 1c, d). Taken together, these results suggest that Ca_V_γ4 is part of the response to glucotoxicity in pancreatic beta cells.

### Ca_V_γ4 is required for glucose-stimulated insulin secretion

**Fig. 2:**
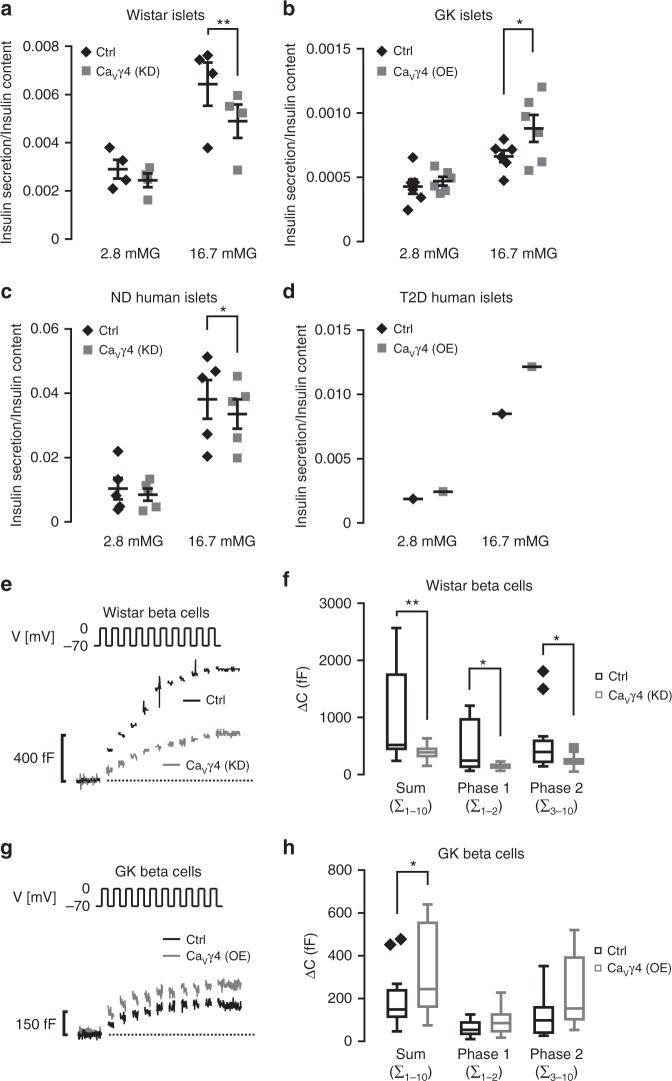
Impact of Ca_V_γ4 on beta-cell function. **a** Glucose-stimulated insulin secretion (GSIS) in Ca_V_γ4-silenced Wistar rat islets. *n* = 4, **p = 0.006. **b** As in **a** but in Ca_V_γ4-overexpressed GK rat islets. *n* = 6, *p = 0.028. **c** As in **a** but in Ca_V_γ4-silenced non-diabetic (ND) human islets. *n* = 5 donors, *p = 0.044. **d** As in **a** but in Ca_V_γ4-overexpressed T2D human islets. *n* = 1 donor (*p* = 0.008 in 16.7mMG group by six technical repeats). **e** Reduced depolarization-evoked (V) exocytosis in Ca_V_γ4-silenced Wistar rat beta cells measured as an increase in membrane capacitance (Δ*C*). **f** A summary of data in **e** presented as Δ*C* evoked by all 10 pulses of the train (Sum), the two first pulses (Phase 1) or the latter eight pulses (Phase 2). *n* = 15 control and 14 Ca_V_γ4-silencing cells, **p = 0.009 (Sum), *p = 0.013 (Phase 1) and *p = 0.028 (Phase 2). **g** As in **e** but rescued exocytosis in Ca_V_γ4-overexpressed GK rat beta cells. **h** Summary of data in **g**. *n* = 15 control and 16 Ca_V_γ4-overexpressing cells, *p = 0.042 (Sum), *p* = 0.07 (Phase 1), and *p* = 0.058 (Phase 2). Data are presented as Mean ± SEM and were analyzed with two-tailed paired (**a**–**c**) or unpaired (**f**, **h**) Student’s *t-*test. OE overexpression, KD knockdown

We next examined if changes in Ca_V_γ4 expression affect beta-cell function. First, glucose-stimulated insulin secretion (GSIS) was markedly inhibited in Ca_V_γ4-silenced Wistar rat islets and non-diabetic human islets (*p* = 0.006 and 0.044, respectively; Fig. 2a, c), and also in INS-1 cells (Supplementary Fig. 2b). Given the reduced Ca_V_γ4 expression under hyperglycemic conditions (Fig. 1), we next overexpressed Ca_V_γ4 to study effects of insulin secretion. This enhanced GSIS in Wistar rat islets (Supplementary Fig. 2a), but more importantly in both diabetic GK rat islets (*p* = 0.028; Fig. 2b) and in T2D human islets (Fig. 2d).

To explore further the impact of Ca_V_γ4 on beta-cell function, we performed capacitance recordings of single-cell exocytosis. In Wistar rat islets, a dramatic reduction of beta-cell exocytosis was detected when silencing Ca_V_γ4 as compared to control cells (389 ± 35 fF vs. 997 ± 206 fF, *p* = 0.009; Fig. 2e, f). A detailed analysis of the readily releasable insulin granules (phase 1) and the sustained phase 2 revealed a decline in both (153 ± 13 fF vs. 453 ± 109 fF, *p* = 0.013 and 236 ± 29 fF vs. 544 ± 126 fF, *p* = 0.028, respectively; Fig. 2f). As expected, Ca_V_γ4 overexpression enhanced overall exocytosis in GK rat beta cells (318 ± 50 fF vs. 190 ± 33 fF, *p* = 0.042; Fig. 2g, h). Successful silencing (by siRNA) or overexpression (by lentivirus) are verified by both qPCR and western blotting in human islets and cell lines (Supplementary Fig. 2c–g).

### Ca_V_γ4 regulates Ca^2+^ influx via L-type Ca_V_ channels

**Fig. 3:**
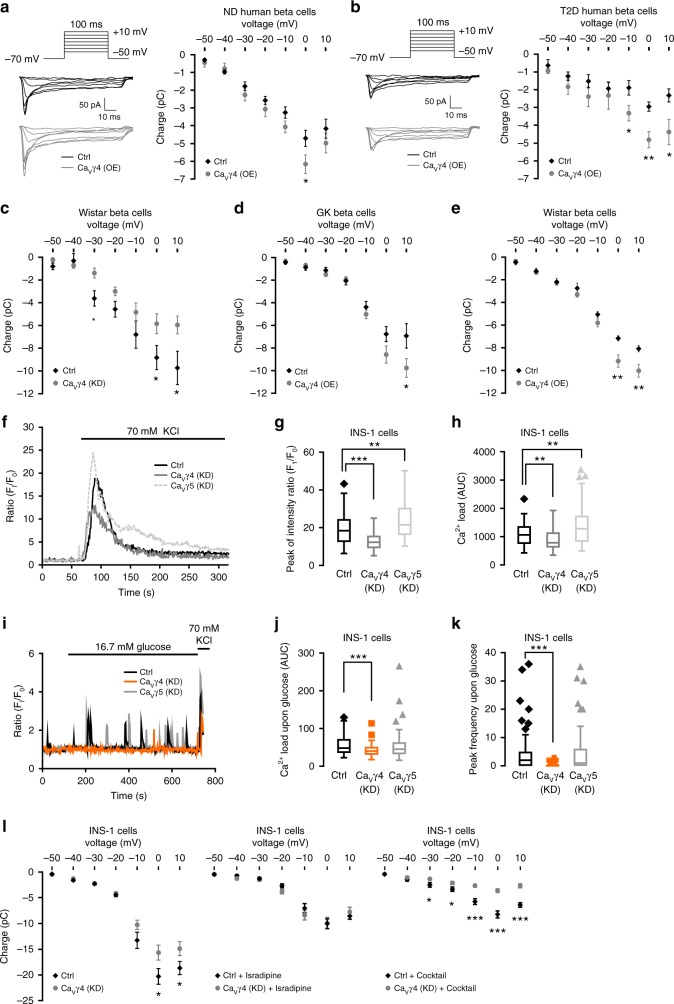
Boost of Ca^2+^ influx in Ca_V_γ4-overexpressed beta cells. **a** Ca^2+^ currents records in Ca_V_γ4-overexpressed non-diabetic (ND) human beta cells. *n* = 12 control and 13 Ca_V_γ4-overexpressing cells (4 donors), *p = 0.048. **b** As in **a** but in T2D human beta cells. *n* = 7 control and 8 overexpressing cells (1 T2D donor), *p = 0.032 (−10 mV), **0.004 (0 mV), *0.028 (10 mV). **c** As in **a** but in Ca_V_γ4-silenced Wistar rat beta cells. *n* = 15 control and 14 silencing cells, *p = 0.040 (0 mV), *0.034 (10 mV). **d** As in **a** but in Ca_V_γ4-overexpressed GK rat beta cells. *n* = 15 control and 14 overexpressing cells, *p = 0.047. **e** As in **a** but in Ca_V_γ4-overexpressed Wistar rat beta cells. *n* = 16 cells each, **p = 0.003 (0 mV), **0.006 (10 mV). **f** Ca^2+^ imaging in control, Ca_V_γ4, or Ca_V_γ5 silenced INS-1 cells. **g** Comparisons of [Ca^2+^]_i_ peak intensity (F_i_/F_0_) in **f**, ***p < 0.001 (Ca_V_γ4), **p = 0.001 (Ca_V_γ5). **h** Integrated Ca^2+^ load (AUC) in **f**, 0–180 s after stimulation. **p = 0.006 (Ca_V_γ4), **p = 0.002 (Ca_V_γ5). *n* = 62 control, 53 Ca_V_γ4- and 61 Ca_V_γ5-silencing cells from three independent experiments for both **g** and **h**. **i** As in **f** but by stimulation of glucose. **j** As in **h** but 0–600 s after glucose stimulation. ***p < 0.001 (Ca_V_γ4) (n.s., Ca_V_γ5). **k** Frequency of [Ca^2+^]_i_ peaks (counted as *F*_i_/*F*_0_ > 1.5) during stimulation. ***p < 0.001 (Ca_V_γ4) (n.s., Ca_V_γ5). *n* = 60 control, 62 Ca_V_γ4- and 61 Ca_V_γ5-silencing cells for both **j** and **k**. **l** Left: As in **a** but in Ca_V_γ4-silenced INS-1 cells. *n* = 32 control and 37 silencing cells, *p = 0.031 (0 mV), *0.049 (10 mV). Middle: in the presence of 2 μM isradipine. *n* = 12 control and 14 silencing cells. Right: or non-L-type channel blocker cocktail (100 nM ω-agatoxin IVA, 50 nM ω-conotoxin GVIA, and 100 nM SNX-482). *n* = 15 control and 21 silencing cells, ***p < 0.001. Data are presented as mean ± SEM and were analyzed with two-tailed unpaired Student’s *t-*test; the significance in **g**, **h**, **j**, **k** was corrected by the Holm–Bonferroni method. OE overexpression, KD knockdown

The Ca_V_γ4-related effects on insulin secretion (Fig. 2a–d, Supplementary Fig. 2a, b) and insulin granule exocytosis (Fig. 2e-h) seen so far might result from enhanced Ca^2+^ influx^2^. Patch clamp offers a direct mode of detection, but it may also be observed in intracellular Ca^2+^ imaging. Therefore, we measured Ca^2+^ currents by patch clamp first, and the representative traces from both condition recordings are shown in Fig. 3a, b, left. Overexpressing Ca_V_γ4 in non-diabetic human beta cells increased whole-cell Ca^2+^ currents compared to control (−6.17 ± 0.52 pC vs. −4.71 ± 0.46 pC, *p* = 0.048; Fig. 3a, right). More strikingly, impaired Ca^2+^ influx in T2D human beta cells was rescued by correcting the reduced Ca_V_γ4 expression levels (−4.82 ± 0.45 pC vs. −2.95 ± 0.26 pC at 0 mV, *p* = 0.004; Fig. 3b, right). Validation in rodent islets showed that silencing Ca_V_γ4 decreased Ca^2+^ influx in Wistar rat beta cells (Fig. 3c), whereas augmented Ca^2+^ influx was recorded in Ca_V_γ4-overexpressing GK and Wistar rat beta cells (Fig. 3d, e).

Next, measurements of intracellular Ca^2+^ concentration ([Ca^2+^]_i_) were done in INS-1 cells. Upon 70 mM KCl stimulation, both the initial [Ca^2+^]_i_ peak and the integrated Ca^2+^ load were markedly lower in Ca_V_γ4 -silenced cells as compared to control cells (12.6 ± 0.6 *F*_i_/*F*_0_ vs. 19.5 ± 1.1 *F*_i_/*F*_0_, *p* < 0.001 and 888.5 ± 50 AUC (area under the curve) vs. 1084.5 ± 48 AUC, *p* = 0.006, respectively; Fig. 3f–h), in agreement with above electrophysiology results. By contrast, Ca_V_γ5 silencing induced the increase of both [Ca^2+^]_i_ peak and Ca^2+^ load (Fig. 3f–h), however, no changes in Ca_V_γ8-silencing cells (Supplementary Fig. 3a, b). Additionally, 16.7 mM glucose, a more physiological stimulus, exerted similar effect on Ca_V_γ4-abolished cells, but not on Ca_V_γ5-silenced cells (Fig. 3i–k). Ca^2+^ currents recorded in Ca_V_γ5- or Ca_V_γ8-silenced INS-1 cells showed no or increased effects, respectively (Supplementary Fig. 3c, d).

Subsequently, we studied the specific involvement of the different pore-forming alpha1 subunits in Ca_V_γ4 mediated Ca^2+^ influx. Intriguingly, when L-type Ca^2+^ currents were pharmacologically inhibited by isradipine (2 μM), an L-type Ca^2+^ channel blocker, silencing of Ca_V_γ4 failed to further suppress Ca^2+^ influx in INS-1 cells (Fig. 3l left, middle). Importantly, when using a cocktail of non-L-type channel blockers (100 nM ω-agatoxin IVA, 50 nM ω-conotoxin GVIA, and 100 nM SNX-482), silencing of Ca_V_γ4 retained its suppressive action on Ca^2+^ influx (Fig. 3l, right).

### Ca_V_γ4 associates with L-type Ca_V_ channel expression

**Fig. 4:**
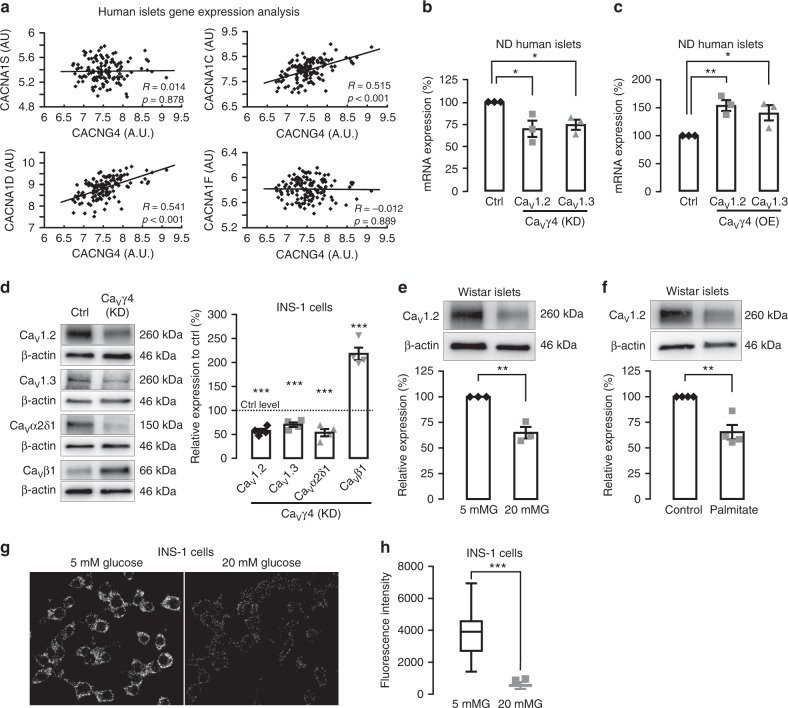
Downregulation of L-type Ca_V_ channels in Ca_V_γ4-silenced beta cells. **a** Correlation of mRNA expression (Microarray) between Ca_V_γ4 (*CACNG4*) and L-type Ca_V_ channels in human islets. *n* = 128 donors. Pearson correlation coefficient (*R*) was tested (*t*-test) and labeled alongside with *p* values. **b** Ca_V_1.2 (*CACNA1C*) and Ca_V_1.3 (*CACNA1D*) mRNA expression (qPCR) in Ca_V_γ4-silenced human islets. *n* = 3 donors, *p = 0.032 (Ca_V_1.2), *p = 0.011 (Ca_V_1.3). **c** As in **b** but in Ca_V_γ4-overexpressed human islets. *n* = 3 donors, **p = 0.005 (Ca_V_1.2), *p = 0.039 (Ca_V_1.3). **d** Ca_V_ channel immunoblotting and means of expression in Ca_V_γ4-silenced INS-1 cells. *n* = 4, ***p < 0.001. **e** Decreased Ca_V_1.2 expression in Wistar islets cultured at 5 or 20 mM glucose (72 h). *n* = 3, **p = 0.003. **f** As in **e** but cultured with 1 mM palmitate (48 h). *n* = 4, **p = 0.003. **g** Interaction between Ca_V_γ4 and Ca_V_1.3 in INS-1 cells treated with 5 or 20 mM glucose (24 h). Visualization by proximity ligation assay as fluorescent spots. **h** Calculation of fluorescent intensity. *n* = 25 cells each, ***p < 0.001. Data are presented as mean ± SEM and were analyzed with two-tailed unpaired Student’s *t-*test; and the significance in **b**, **c** was corrected by the Holm–Bonferroni method. OE overexpression, KD knockdown

Next, we explored how Ca_V_γ4 associates with the different L-type Ca_V_ channels. First, human islets microarray data showed a strong positive correlation between Ca_V_γ4 and L-type Ca_V_ subunits known to be involved in human insulin secretion, Ca_V_1.2 (*CACNA1C*), and Ca_V_1.3 (*CACNA1D*), whereas it displayed no correlation between Ca_V_γ4 and Ca_V_1.1 (*CACNA1S*) or Ca_V_1.4 (*CACNA1F*) (Fig. 4a). Interestingly, silencing Ca_V_γ4 resulted in downregulation of both Ca_V_1.2 and Ca_V_1.3 gene expression in non-diabetic human islets (*p* = 0.032 and 0.011, respectively; Fig. 4b). Meanwhile, the Ca_V_1.2 and Ca_V_1.3 mRNA levels were elevated by overexpressing Ca_V_γ4 in non-diabetic human islets (*p* = 0.005 and 0.039, respectively; Fig. 4c), as well as in T2D human islets (Supplementary Fig. 4a). Similarly, depleting Ca_V_γ4 reduced protein levels of Ca_V_1.2 and Ca_V_1.3 in INS-1 cells, and also the levels of Ca_V_ alpha2delta (Ca_V_α_2_δ1), but increased the protein expression of beta subunits (Ca_V_β1) (*p*<0.001 for all; Fig. 4d); findings correlating with changes in mRNA (Supplementary Fig. 4b). Additionally, Ca_V_1.2 expression was clearly reduced in glucose- or palmitate-challenged Wistar rat islets (*p* = 0.003 both; Fig. 4e, f) and INS-1 cells (Supplementary Fig. 4c). Intriguingly, 24-h treatment with 20 mM glucose reduced the direct spatial interaction between Ca_V_γ4 and Ca_V_1.3 detected by proximity ligation assay (*p*<0.001; Fig. 4g, h). This dissociation, under hyperglycemic condition, could reflect an altered affinity between the subunits or simply be due to the lower expression of Ca_V_γ4 (see Fig. 1). For Ca_V_1.2, we failed to detect interaction, the significance of which remains uncertain.

### MafA mediates Ca_V_γ4 expression in beta cells

**Fig. 5:**
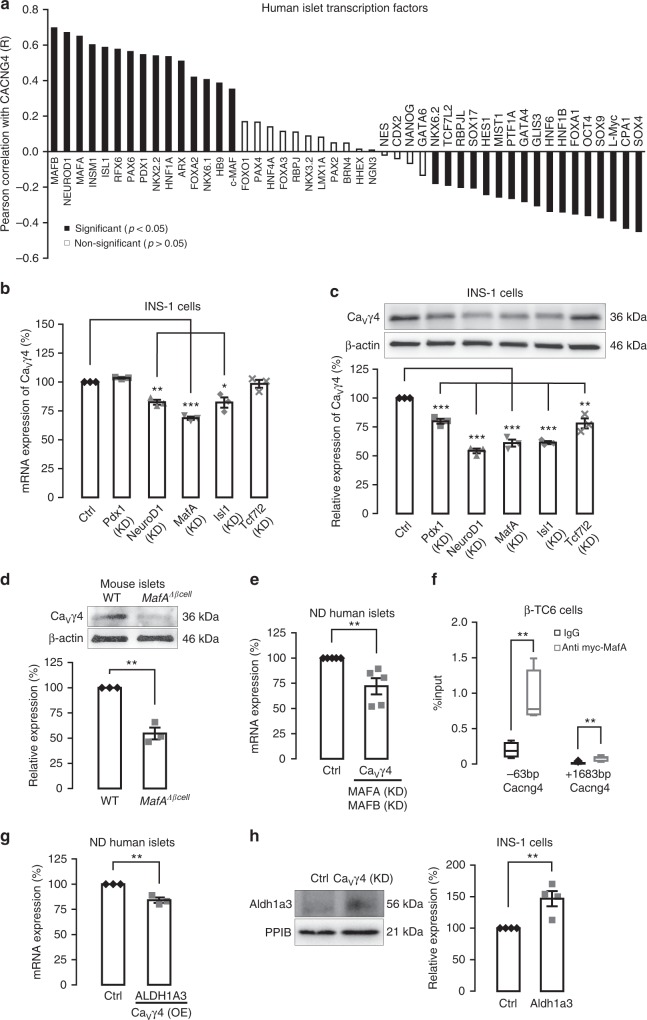
Ca_V_γ4 is downregulated by silencing of the transcription factor MafA. **a** Rank of Pearson correlation coefficient (*R*) (tested by *t*-test) calculated by mRNA expression (Microarray, human islets) between Ca_V_γ4 (*CACNG4*) and the transcription factors known for pancreas development. *n* = 128 donors. **b** Ca_V_γ4 mRNA expression in Pdx1, NeuroD1, MafA, Isl1, or Tcf7l2 silenced INS-1 cells. *n* = 3, **p = 0.001 (NeuroD1), ***p < 0.001 (MafA), *p = 0.016 (Isl1). **c** As in **b** but Ca_V_γ4 protein expressions were measured. *n* = 3, ***p < 0.001 (Pdx1, NeuroD1, MafA, Isl1), **p = 0.007 (Tcf7l2). **d** Decreased Ca_V_γ4 expression in *MafA*^*Δβcell*^ islets. *n* = 3 mice each, **p = 0.001. **e** Ca_V_γ4 mRNA expression was reduced in MAFA and MAFB double-silenced human islets. *n* = 5 donors, **p = 0.005. **f** qPCR amplification of Ca_V_γ4 (Cacng4) regulatory sequences after immunoprecipitation of chromatin transfected with myc-tagged MafA in βTC6 cells. Data are presented as %input. *n* = 4 (−63 bp) and 6 (+1683 bp), respectively, **p < 0.01. **g** *ALDH1A3* mRNA expression in Ca_V_γ4-overexpressed human islets. *n* = 3 donors, **p = 0.004. **h** Aldh1a3 protein expression in Ca_V_γ4-silenced INS-1 cells (96 h). *n* = 4, **p = 0.008. Data are presented as mean ± SEM and were analyzed with two-tailed unpaired Student’s *t-*test, and the significance in **b**, **c** was corrected by the Holm–Bonferroni method. WT wild type, Ctrl control, KD knockdown

Given the surprisingly important role of Ca_V_γ4 in beta cells, the factors that regulate Ca_V_γ4 expression are of special interest. Furthermore, pathophysiological alterations observed above when depleting Ca_V_γ4 are reminiscent of a dedifferentiated diabetic beta-cell phenotype. We therefore explored genes involved in pancreas or beta-cell development, for co-regulation with Ca_V_γ4 in human islets microarray (Fig. 5a). These data revealed a strong positive correlation between Ca_V_γ4 and the important beta-cell differentiation transcription factors *NEUROD1*, *MAFA*, *ISL1*, and *PDX1* (Supplementary Fig. 5a). To determine the causality of this correlation, Pdx1, NeuroD1, MafA, Isl1, and Tcf7l2 were silenced in INS-1 cells, respectively (successful silencing has been proved previously^25^), with MafA silencing having the largest effect on Ca_V_γ4 mRNA expression (***p < 0.001; Fig. 5b). Also Ca_V_γ4 protein levels were profoundly reduced after MafA silencing (***p < 0.001; Fig. 5c). Given that MafA is predominantly expressed in adult rodent beta cells as a selective beta-cell marker^26^, and is found to be capable of reprogramming acinar cells to beta-like cells^20^; moreover, Pdx1, NeuroD1, and Isl1 are also expressed in other types of endocrine cells in islets^27,28^. Against this backdrop, we further explored the effects of MafA on Ca_V_γ4. In beta-cell-specific MafA knockout mice (*MafA*^*Δβcell*^), Ca_V_γ4 protein levels were strongly reduced in vivo (*p* = 0.001; Fig. 5d). Confirmation in vitro showed that Ca_V_γ4 expression was notably decreased in MAFA/MAFB double-silenced human islets (*p* = 0.005; Fig. 5e). MAFA ablation alone failed to induce an alteration on Ca_V_γ4 expression (Supplementary Fig. 5b). This we attribute to the fact that human beta cells express both MAFA and MAFB while only MafB is detected in adult mouse alpha cells^29^; which suggests a possible compensatory effect of MAFB in human beta cells. Furthermore, chromatin immunoprecipitation analysis disclosed two binding sites of MafA to Ca_V_γ4 promoter region (**p < 0.01; Fig. 5f), indicating that Ca_V_γ4 is directly regulated by MafA.

Downregulation of Ca_V_γ4 results in beta-cell dedifferentiation rather than other dysfunctions since the phenotype of Ca_V_γ4-silenced beta cell as detailed above remains unaltered. Recently, a new beta-cell dedifferentiation marker aldehyde dehydrogenase1a3 (Aldh1a3) was found to be highly enriched in dedifferentiated islets^30^ and suggested marker of dedifferentiated beta cells in human type-2 diabetic islets^22^. Here, *ALDH1A3* gene expression was decreased in Ca_V_γ4-overexpressed non-diabetic human islets (*p* = 0.004; Fig. 5g), whereas protein levels were markedly upregulated in Ca_V_γ4-silenced INS-1 cells (*p* = 0.008, Fig. 5h). The results are perfectly consistent with data on co-expression of *ALDH1A3* with *CACNG4* by human islets microarray data (Supplementary Fig. 5c). Additionally, silencing Ca_V_γ4 failed to induce any alterations in cleaved Caspase-3 and P21 expression, cell viability (MTT) or apoptosis (7-AAD staining) (see Supplementary Fig. 5d–f), indicating beta-cell health is not influenced by Ca_V_γ4 expression.

### Reduced Ca^2+^ currents in *MafA*^*Δβcell*^ beta cells

**Fig. 6:**
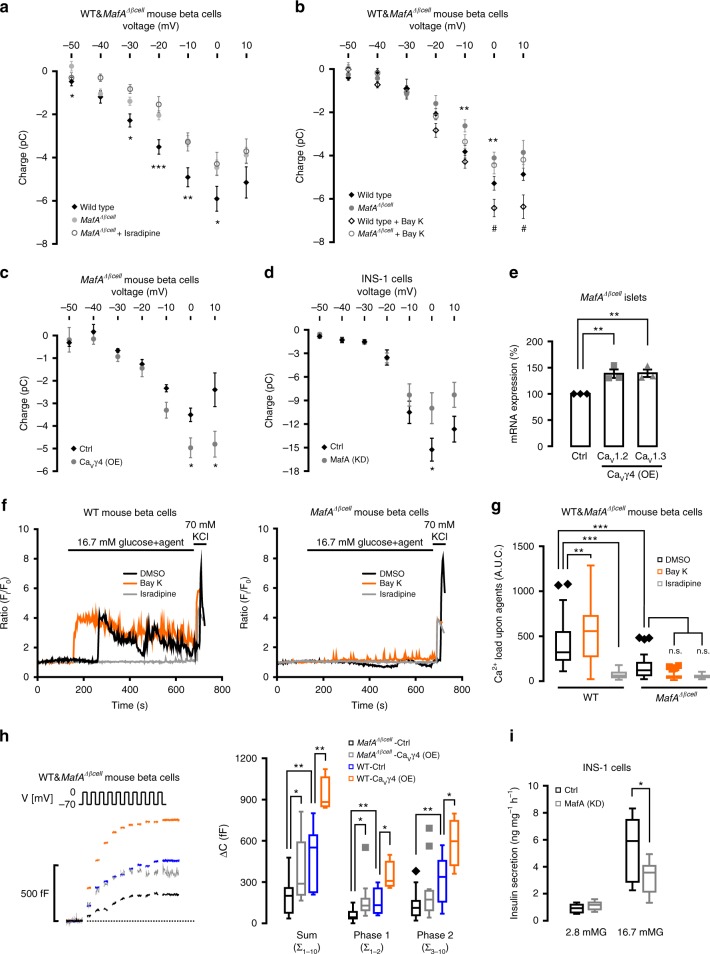
Reduced Ca^2+^ currents and GSIS by silencing of MafA. **a** Whole-cell Ca^2+^ charge–voltage relations in beta cells from wild-type mice, *MafA*^*Δβcell*^ and *MafA*^*Δβcell*^ in the presence of 2 μM isradipine. *n* = 13, 15, and 7 cells, respectively, *p* = 0.024*, 0.020*, 0.004**, 0.036* and *p* < 0.001*** for −50, −30, −10, 0 , and −20 mV, respectively. **b** As in **a** but in wild type and *MafA*^*Δβcell*^ beta cells in the absence (*n* = 17 wild type and 16 mutant cells) or presence (*n* = 19 wild type and 21 mutant cells) of 300 nM Bay K8644. **p = 0.006 (−10 mV), **p = 0.008 (0 mV) for wild type vs*. MafA*^*Δβcell*^; ^#^*p* = 0.036 (0 mV), ^#^*p* = 0.021 (10 mV) for wild type in the absence or presence of Bay K8644. **c** As in **a** but in Ca_V_γ4-overexpressed *MafA*^*Δβcell*^ beta cells. *n* = 5 control and 8 overexpressing cells, *p = 0.035 (0 mV), *p = 0.026 (10 mV). **d** As in **a** but in MafA silenced INS-1 cells. *n* = 15 control and 18 silencing cells, *p = 0.046. **e** Ca_V_1.2 and Ca_V_1.3 mRNA expression in Ca_V_γ4 overexpressed *MafA*^*Δβcell*^ islets. *n* = 3, **p = 0.009 (Ca_V_1.2), **p = 0.005 (Ca_V_1.3). **f** Intracellular Ca^2+^ concentration ([Ca^2+^]_i_) measured in wild type (left) and *MafA*^*Δβcell*^ (right) beta cells by stimulation of 16.7 mM glucose in the presence of DMSO, Bay K8644 (300 nM), or isradipine (2 μM) for 600 s. **g** Ca^2+^ load in **f**, 0–600 s after stimulation. *n* = 52 (wild-type-DMSO), 65 (wild-type-BayK), 45 (wild-type-isradipine), 50 (*MafA*^*Δβcell*^-DMSO), 52 (*MafA*^*Δβcell*^-BayK), and 34 (*MafA*^*Δβcell*^-isradipine) cells; **p < 0.01, ***p < 0.001, n.s. not significant. **h** Increased exocytosis in Ca_V_γ4-overexpressed *MafA*^*Δβcell*^ beta cells measured as Δ*C* (left), and the summary of data (right). *n* = 13 (*MafA*^*Δβcell*^-Ctrl), 13 (*MafA*^*Δβcell*^-Ca_V_γ4(OE)), 8 (wild-type-Ctrl), and 4 (wild-type-Ca_V_γ4(OE)) cells, *p < 0.05, **p < 0.01. **i** Reduced GSIS (16.7 mM) in MafA silenced INS-1 cells. *n* = 9, *p = 0.021. Data are presented as mean ± SEM and were analyzed with two-tailed unpaired Student’s *t-*test (**a**–**e**, **h**, **i**) and one-way ANOVA with Tukey’s multiple comparisons test (**g**); and the significance in h was corrected by the Holm–Bonferroni method. WT wild type, Ctrl control, KD knockdown, OE overexpression

We next tested the hypothesis as suggested above to the effect that MafA controls Ca_V_γ4 expression, which in turn has consequences for L-type Ca_V_ channels specific Ca^2+^ influx and function of beta cells. In support of this, Ca^2+^ currents were reduced in *MafA*^*Δβcell*^ beta cells. Interestingly, and in accord with the hypothesis, the L-type Ca^2+^ channel blocker isradipine (2 μM) failed to affect Ca^2+^ influx (Fig. 6a). Conversely, the L-type Ca^2+^ channel agonist Bay K8644 (300 nM) potentiated Ca^2+^ influx in wild-type mouse beta cells, while being ineffective in MafA-depleted beta cells (Fig. 6b). Further support came from the observation that overexpressing Ca_V_γ4 in *MafA*^*Δβcell*^ islets resulted in elevated beta-cell Ca^2+^ influx (Fig. 6c). In addition, the role of MafA in Ca^2+^ signaling was confirmed in INS-1 cells (Fig. 6d). As expected, re-introducing Ca_V_γ4 in *MafA*^*Δβcell*^ islets raised both Ca_V_1.2 and Ca_V_1.3 mRNA expression (*p* = 0.009 and 0.005, respectively; Fig. 6e), as well as in MAFA silenced human EndoC cells (Supplementary Fig. 6). Similar to data in Fig. 6a, b, Ca^2+^ imaging in *MafA*^*Δβcell*^ and wild-type mouse beta cells exposed to Bay K8644 (300 nM) or isradipine (2 μM) (Fig. 6f, g) strongly substantiated the idea that L-type Ca^2+^ channels are downstream target of MafA, with impacting on Ca^2+^ influx in beta cells. Furthermore, we recorded an almost 50% rescue of exocytosis (particularly the readily releasable pool), in Ca_V_γ4-overexpressing *MafA*^*Δβcell*^ beta cells, restoring exocytosis at levels similar to that in wild-type beta cells (Fig. 6h). Finally, reduced GSIS was observed after silencing MafA in INS-1 cells (Fig. 6i).

**Fig. 7:**
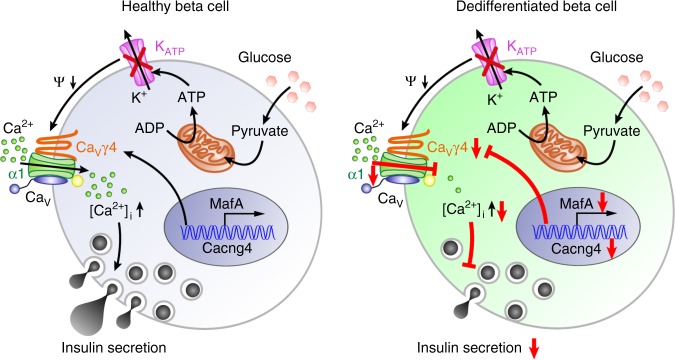
Schematic of regulation cascade from MafA through Ca_V_γ4 to insulin secretion in beta cell. Compared with healthy beta cell, dedifferentiated beta cell caused by T2D or glucotoxicity results in reduced MafA expression, which leads to the downregulation of its direct downstream target Ca_V_γ4. Decreased Ca_V_γ4 expression then diminishes L-type Ca_V_ channels expression with consequent preventing of Ca^2+^ influx and in turn blunting of GSIS

These results suggest that MafA controls Ca^2+^ influx via an effect involving Ca_V_γ4 and L-type Ca_V_ channels, which eventually initiate insulin release. However, this pathway is affected at multiple sites by glucotoxicity and in diabetic conditions leading to a dedifferentiated and diabetes-prone phenotype (Fig. 7).

## Discussion

Ca_V_γ subunits were first isolated in guinea pig skeletal muscle in the form of a complex with L-type Ca^2+^ channels and Ca_V_γ1 subunits^31^. Ca_V_γ2, Ca_V_γ3, Ca_V_γ4, and Ca_V_γ8 subunits have later been shown to associate with AMPA receptors in neurons^32,33^, but Ca_V_γ4 and Ca_V_γ8 are demonstrated to physically interact with the cardiac L-type Ca^2+^ channel Ca_V_1.2 (ref. ^9^). The rationale for focusing on Ca_V_γ4 stems from it being the only differentially expressed Ca_V_γ subunit in human diabetic islets (Fig. 1a). Furthermore, Ca_V_γ4 is also downregulated in hyperglycemic and T2D animal models, GK rat and *db/db* mouse islets^34^ as well as by environmental stress in the form of high glucose and palmitate in human islets, Wistar rat islets, and clonal cells (Fig. 1). Interestingly, Ca_V_γ4 expression is unaffected in Akita mouse islets, a model of ER stress, may suggests that Ca_V_γ4 action occurs earlier in glucotoxicity. Ca_V_γ4 is involved in regulation of L-type Ca^2+^ channel gene expression, as demonstrated here in human islets for both Ca_V_1.2 and Ca_V_1.3 (Fig. 4b, c, Supplementary Fig. 4a), as well as on protein levels in INS-1 cells (Fig. 4d). Accordingly, Ca_V_γ4 correlated evidently with Ca_V_1.2 and Ca_V_1.3 in human islets microarray analysis (Fig. 4a), and exhibited a direct interaction with Ca_V_1.3 in INS-1 cells (Fig. 4g, h). By contrast, the impact of Ca_V_γ4 on expression of the other L-type channels, the predominantly skeletal Ca_V_1.1 and retinal Ca_V_1.4 (ref. ^3^), were very weak (Fig. 4a). Interestingly, Ca_V_γ4 is expressed throughout the entire cell volume in human beta cells (Fig. 1b), which differs from previous observations by electron microscopy that Ca_V_γ4 locates close to the plasma membrane^35^. The demonstrated direct interaction between Ca_V_γ4 and Ca_V_1.3 (Fig. 4g, h) suggests effects on modulating Ca^2+^ influx by, e.g., facilitating L-type Ca^2+^ channel trafficking, internalization, and degradation, but also potential functions completely unrelated to Ca^2+^ homeostasis, which will be explored in future. Be that as it may, Ca_V_γ4 clearly determines beta-cell functionality by enhancing Ca^2+^ entry through L-type Ca^2+^ channels. The specificity of Ca_V_γ4 for modulating L-type Ca^2+^ currents is demonstrated by the fact that L-type Ca^2+^ channel blocker isradipine was ineffective in Ca_V_γ4-silenced cells. In agreement with this finding, Ca_V_γ4 did not influence the amplitude of non-L-type Ca^2+^ currents (Fig. 3l). Interestingly, the reduced Ca^2+^ influx and exocytosis in T2D human and GK rat beta cells were rescued when overexpressing Ca_V_γ4 (Figs. 3b, d and 2g, h), and insulin secretion was also partly recovered in T2D human and GK rat islets (Fig. 2b, d). In summary, Ca_V_γ4 plays a more important role than anticipated for Ca^2+^ signaling in beta cells and appears to be a central player in the regulation of insulin secretion (Fig. 2 and Supplementary Fig. 2). For future studies, the molecular structural mechanisms by which Ca_V_γ4 connects to the L-type alpha1 subunits will be an important issue to resolve.

Ca_V_ channels have dual roles and are key for regulation of gene expression and beta-cell differentiation^4,36,37^. They generate Ca^2+^ signals that activates specific Ca^2+^-dependent pathways that control gene expression^36–39^. During the process of differentiation, transcription factors regulate Ca_V_ channel subunits expression. This generates a controlled temporal pattern of activating Ca^2+^ signals for the above-mentioned transcriptional effects^40^. In beta cells, the transcription factor MafA is responsible for beta-cell-specific expression of insulin and also reprograms adult pancreatic acinar cells into beta-like cells by incompletely elucidated mechanisms^20^. Importantly, the nuclear levels of MafA are drastically and specifically reduced in oxidative stressed beta cell lines, *db/db* mouse, and T2D human islets, whereas Pdx1, Isl1, and NeuroD1 are unaffected under such conditions^41^.

Ca^2+^ transients in the cytosol have been implicated in initiating differentiation of mesenchymal cells^42^. These transients originate either from intracellular stores or from influx of extracellular Ca^2+^ through Ca_V_ channels in response to membrane depolarization. Ca_V_γ4 is highly expressed in the fetal brain, as well as in the subpopulation of differentiating myoblasts and has been suggested to play an important role in regulating intracellular Ca^2+^ transients^18^. The present study shows that in human islets Ca_V_γ4 is correlated with many transcription factors known in pancreas development or endocrine cell differentiation (Fig. 5a). Notably, we here provide evidence for Ca_V_γ4 being one of the downstream targets of MafA and one that has profound impact on the mature beta-cell phenotype. First, MafA exhibits a strong positive correlation to Ca_V_γ4 in human islets (Supplementary Fig. 5a). Second, expression of Ca_V_γ4 is markedly reduced in *MafA*^*Δβcell*^ islets, MAFA and MAFB double-depleted human islets, as well as MafA silenced INS-1 cells, with equally strong alteration of voltage-gated L-type Ca^2+^ currents, and in turn, [Ca^2+^]_i_, exocytosis and insulin release (Figs. 5 and 6). Most importantly, MafA directly binds to the promoter region of Ca_V_γ4 (Fig. 5f). In addition, the fact that MafA levels are markedly reduced in beta cells upon chronic oxidative stress as glucose toxicity develops is well established^41,43,44^. Collectively, these results demonstrate the connection between glucotoxicity, differentiation, gene expression, and functional consequences (Ca^2+^ signaling, secretion) for the beta-cell. In the human microarray data set, Ca_V_γ4 exhibited the strongest correlation with *MAFB* among all transcription factors (Fig. 5a). Given that in rodents MafB is exclusively expressed in adult alpha-cell, whereas MafA is necessary for adult beta-cell function, we hence focused on the effect of MafA on Ca_V_γ4 in the present study. However, MafB is crucial for both alpha- and beta-cell differentiation during islet development and has also been detected in adult human beta cells^20,26,29^, a future thorough investigation of the interactions between MafB and Ca_V_γ4 is warranted. Besides MafA, in another high-throughput CHIP-on-chip data set of global Pdx1 occupancy revealed that Pdx1 also directly binds to the promoter region of Cacng4 (Ca_V_γ4) in mouse Min6 cells^45^. Moreover, MafA was strongly downregulated in both acute Pdx1-deficient Min6 cells and chronic Pdx1 heterozygous (*Pdx1*^*+/−*^) mouse islets^45^. This taken together with our data to the effect that silencing of MafA (and/or Pdx1) results in reduced Ca_V_γ4 protein levels (Fig. 5c), strongly suggests that Ca_V_γ4 is a direct target of MafA (and Pdx1) in regulating beta-cell differentiation. Interestingly, Ca_V_γ4 expression was also regulated by NeuroD1, Isl1, and Tcf7l2 in the current study (Fig. 5b, c). Here we hypothesize that the transcription factors interact with the promotor of Ca_V_γ4 gene by a direct binding (NeuroD1, Isl1) or an indirect impact through MafA or Tcf7l2 (ref. ^25^). To identify the underlying mechanisms, future studies are warranted on how Ca_V_γ4 and other Ca_V_ channels are transcribed, translated, and transported to plasma membrane.

Aldh1a3 has long been recognized as a standard marker of cancer precursor cells^46^, but was recently reported to be elevated in beta-cell dedifferentiated animal model, and it is now regarded an endocrine progenitor cell marker^22,30^. This agrees with data in the current study to the effect that alteration of Ca_V_γ4 expression leads to changes in Aldh1a3 expression in both human islets and INS-1 cells (Fig. 5g, h, Supplementary Fig. 5c), while not affecting cell viability, apoptosis, or proliferation (Supplementary Fig. 5d–f).

In conclusion, we demonstrate an essential role of the Ca_V_γ4 subunit for maintaining the normal pancreatic beta-cell phenotype in humans. This resonates well with the emerging view that Ca_V_ channels exert long-term effects on gene expression and RNA editing^11,37^. Ca_V_γ4 is part of the MafA pathway that controls the final stage of beta-cell differentiation, and is suppressed in vivo and in vitro by hyperglycemic stress. Taken together, these results indicate a fundamental role for Ca_V_-generated signals in long-term regulation of the beta-cell functional status in health and disease.

## Methods

### Human islets

Human pancreatic islets were obtained provided through collaboration between Human Tissue Laboratory within Lund University and the Nordic Network for Clinical Islet Transplantation (Uppsala University, Sweden). The human islets (70–90 % purity) had been cultured in CMRL 1066 (ICN Biomedicals, Costa Mesa, CA) supplemented with 10 mM HEPES, 2 mM l-glutamine, 50 μg ml^−1^ gentamicin, 0.25 μg ml^−1^ fungizone (Gibco, BRL, Gaithersburg, MD), 20 μg ml^−1^ ciprofloxacin (Bayer Healthcare, Leverkusen, Germany), and 10 mM nicotinamide at 37 °C (5% CO_2_) for 1–5 days prior to the arrival in the laboratory. The islets were then handpicked under a stereomicroscope. All procedures were approved by the ethics committees at Uppsala and Lund Universities. See Supplementary Table 1 for the characteristics of human islet donors.

### Animals

Adult beta-cell MafA deletion mutant mice were generated using the Cre-*loxP*-mediated recombination system. The *MafA*^*fl/fl*^, *MafA*^*−/−*^, and *RIP (rat insulin promoter)-cre* mouse lines have been generated previously^47^. The *RIP-Cre* mouse line does not contain the human growth hormone minigene, which is in contrast to other commonly used pancreas-specific Cre deleter lines, which exhibit defects associated with the ectopic expression of human growth hormone in islets. In brief, a conditional *MafA* allele was generated earlier, and a *MafA* deletion in adult beta cells was achieved by crossing *MafA*^*fl/fl*^ mice with mice expressing Cre Recombinase under the rat insulin promoter (RIP-cre). *RIP-cre* mice were referred to as *MafA*^*Δβcell*^ mutants and resulted in complete loss of MafA expression, impaired glucose clearance after an intraperitoneal glucose challenge^47^. Mice were maintained on a mixed C57BL/6 and Sv129 background.

Wistar and GK male rats (Charles River Laboratories, Wilmington, MA) 6–11 week of age was used. Blood glucose at termination was measured to ensure the normoglycemic level for Wistar and hyperglycemia for GK. Adult *db/db* and control (C57/bl) mice (Janvier Laboratory, France), and Akita (*Ins2*^*+/−*^) and wild-type (*Ins2*^*+/+*^) male littermates (Jackson laboratories, stock number 003548) were used (7–13 weeks) for the indicated experiment. All animal experimentation was conducted in accord with accepted standards of humane animal care and approved by the local ethics committee.

### Human pancreatic islets microarray data

Human islets whole transcript microarray analysis was performed using GeneChip Human Gene 1.0 ST and processed with the standard Affymetrix protocol. The array data were then summarized and normalized with the Robust Multi-array Analysis (RMA) method using the oligo package from BioConductor. Batch correction was done with COMBAT function from SVA package from BioConductor. Annotation was done using annotate package from BioConductor and hugene10sttranscriptcluster.db annotation data. Probesets were only kept if they matched uniquely to a gene in the latest hg19 human genome assembly. If more than one probeset matched a gene, one probeset at random was chosen in order to have only one probeset per gene. Finally, only probesets (or genes) mapped to the autosomes were kept^24^.

### Cell culture

INS-1 832/13 cells (kindly donated by Dr. C. B. Newgaard, Duke University, USA) were cultured in RPMI-1640 containing 11.1 mM d-glucose and supplemented with 10% fetal bovine serum, 100 U ml^−1^ penicillin (Gibco), 100 μg ml^−1^ streptomycin (Gibco), 10 mM *N*-2 hydroxyethylpiperazine-*N*′-2-ethanesulfonic acid (HEPES), 2 mM glutamine, 1 mM sodium pyruvate, and 50 μM β-mercaptoethanol (Sigma), at 37 °C in a humidified atmosphere containing 95% air and 5% CO_2_.

EndoC-βH1 cells (EndoCells, Paris, France) were maintained in a culture medium containing: DMEM (5.6 mM glucose), 2% BSA fraction V (Roche), 10 mM nicotinamide (Merck), 50 µM 2-mercaptoethanol, 5.5 µg ml^−1^ transferrin, 6.7 ng ml^−1^ sodium selenite (Sigma), 100 U ml^−1^ penicillin, and 100 µg ml^−1^ streptomycin (PAA Laboratories). For β-TC6 cell culture see below.

### Islets isolation and preparation

Intact primary pancreatic islets were isolated by retrograde injection of a collagenase solution via the pancreatic duct from fed rats and 2-month old wild type and mutant mice, and were handpicked under a stereomicroscope at room temperature. The isolated islets were kept in RPMI-1640 medium for culture, but substituted with 5 mM d-glucose (for rats) or 10 mM d-glucose (for mice) and lack of β-mercaptoethanol. All indicated experiments were conducted on freshly isolated islets.

To preform electrophysiology and Ca^2+^ imaging experiments, the isolated islets were dispersed into single cells using Ca^2+^-free buffer and allowed to adhere in 35-mm Petri dishes (Nunc, Thermo Scientific) coated by poly-l-lysine (Sigma) and cultured as above.

### siRNA transfection

INS-1 832/13 cells were seeded 1 day before transfection. Thirty nanomolar RNA interference oligonucleotides (Ambion, USA) or Negative Control #1 (Ambion, USA) were applied to silence target genes. Transfection reagent (Dharmafect, Thermo Scientific, USA) was used. For primary human and rat islets, reverse transfection was performed to reach a faster and high-throughput transfection. Islets and siRNA-lipid complexes were prepared on the same day with Lipofectamine RNAiMAX (Invitrogen, USA) as transfection reagent. Transfection efficiency was measured by real-time PCR, western blotting, and visualized by BLOCK-iT Alexa Fluor Red Fluorescent Control (Invitrogen, USA).

### Lentiviral transfection

Cacng4 or control (lentiviral particles without targeting any specific region) plasmids cloned in Lentiviral based shuttle vectors with mGFP tagged (ORIGENE, USA) were transformed into *E. coli* on LB-agar plates supplemented with 34 μg ml^−1^ chloramphenicol. Amplified plasmids were purified with QIAGEN Plasmid Midi Kit (QIAGEN, USA). Lentiviral vectors were produced through transfection into HEK293T cells, and harvested followed by concentration and titration by service from Lund University Stem Cell Center Vector Unit. Primary Human, rat or mouse islets and EndoC cells were transfected with Cacng4 or control lentiviral vectors by directly adding into the culture medium under the calculation of 1 multiplicity of infection (MOI) for 72 h (change with fresh medium after 48 h transfection, and incubate another 24 h), followed by specific experiments indicated in results. Successful transfection was validated by qPCR, western blotting, and visualized though UV light under microscopy.

### Insulin secretion

Transfected INS-1 832/13 cells were firstly washed twice with 1 ml pre-warmed Secretion Assay Buffer (SAB), pH 7.3 (114 mM NaCl, 4.7 mM KCl, 1.2 mM KH_2_PO_4_, 1.16 mM MgSO_4_, 20 mM HEPES, 2.5 mM CaCl_2_, 25.5 mM NaHCO_3_, and 0.2% bovine serum albumin) supplemented with 2.8 mM glucose. The cells were then preincubated for 2 h in 1 ml new SAB with 2.8 mM glucose at 37 °C. Afterwards, insulin secretion was induced by static incubation with either 2.8 or 16.7 mM glucose for 1 h in 0.5 ml SAB, respectively. Secreted insulin was measured through the insulin Coat-a-Count RIA (Siemens Healthcare Diagnostics, IL, USA) by running on the 2470 WIZARD2 Automatic Gamma Counter (PerkinElmer, USA) or through high range rat insulin ELISA kit (Mercodia, Sweden), and normalized according to total protein content per well. Protein content was determined by Pierce BCA protein assay kit (Thermo Scientific, USA).

Transfected primary human or rat islets were preincubated with Krebs Ringer bicarbonate buffer (KRB), pH 7.4 (120 mM NaCl, 4.7 mM KCl, 2.5 mM CaCl_2_, 1.2 mM KH_2_PO_4_, 1.2 mM MgSO_4_, 25 mM NaHCO_3_, 10 mM HEPES, and 1 mg ml^−1^ BSA) for 30 min containing 2.8 mM glucose at 37 °C. Each incubation vial contained 12 size-matched islets in 1 ml KRB buffer and was treated with 95% O_2_ and 5% CO_2_ to obtain constant pH and oxygenation. After preincubation, the buffer was changed to 1 ml KRB buffer supplemented with either 2.8 or 16.7 mM glucose. The islets were then incubated for 1 h at 37 °C in a metabolic shaker (30 cycles per min). Immediately after incubation an aliquot of the medium was removed for analysis of secreted insulin and the islets were homogenized for measurement of insulin content using rat insulin RIA kit (Merck Millipore, Germany) or human insulin ELISA kit (Mercodia, Sweden). For each individual, measurements were performed in 6–8 vials per condition.

### Electrophysiology

Whole-cell capacitance and whole-cell Ca^2+^ current measurements were performed in INS-1 832/13 cells or primary human, rat or mouse beta cells using HEKA EPC10 patch-clamp amplifiers with the software suite Pulse + X-Chart Extension (version 8.6 or later; HEKA, Lambrecht-Pfalz, Germany)^48^. Primary islet cells or INS-1 832/13 cells were seeded in Nunc plastic Petri dishes and were used for experiments the following day. The bath was continuously perfused with extracellular solution containing 118 mM NaCl, 20 mM tetraethylammonium chloride, 5.6 mM KCl, 2.6 mM CaCl_2_, 1.2 mM MgCl_2_, 5 mM HEPES, and 5 mM glucose (pH 7.4 with NaOH), and the temperature maintained at 32 °C. The pipette (intracellular) solution consisted of 125 mM Cs-glutamate, 10 mM CsCl, 10 mM NaCl, 1 mM MgCl_2_, 5 mM HEPES, 3 mM Mg-ATP, 0.1 mM cAMP, and 0.05 mM EGTA (pH 7.2 with CsOH). 0.05 mM EGTA was replaced by 1.5 mM Bapta in the pipette solution for Ca^2+^ currents recording. L-type Ca^2+^ channel blocker isradipine (2 μM, Sigma), potentiation Bay K8644 (300 nM, Sigma), P/Q-type channel blocker ω-agatoxin IVA (100 nM, Alomone labs), N-type channel blocker ω-conotoxin GVIA (50 nM, Alomone labs), and R-type channel blocker SNX-482 (100 nM, Alomone labs) were added as indicated in text or figures. The whole-cell configuration was used in voltage-clamp mode and pipettes had an average resistance of ≈5.5 MΩ. Beta- and alpha-cells were identified by the virtue of Na^+^ channel inactivation features, with beta cells exhibiting half-maximal Na^+^ channel inactivation at membrane potentials lower than −100mV and alpha-cells greater than −100mV^4^.

### Quantitative PCR

Total RNA was extracted using the RNAeasy Kit (QIAGEN, Germany) after transfection. One microgram of RNA was used for cDNA synthesis. Primers of housekeeping genes HPRT1, B2M, and POLR2A (TaqMan Gene Expression, USA) and genes of interest (TaqMan Gene Expression) which tagged FAM dyes were used for amplification detection. The real-time PCR was carried out as follows: 50 °C for 2 min, 95 °C for 10 min, 40 cycles of 95 °C for 15 s, and 60 °C for 1 min by running on a ViiA 7 Real-Time System (Applied Biosystems) with total reaction mixture (10 μl) consisting of 5 μl TaqMan Universal PCR Master Mix (Applied Biosystems), 2.5 μl 4× primer, and cDNA.

### Western blotting

INS-1 832/13 cells or primary islets were homogenized in ice cold RIPA buffer containing complete protease inhibitor (Roche) by vortex or shaking on ice for 30 min. Supernatant was collected by centrifugation (10,000 × *g*, 15 min, 4 °C). Extracted total protein content was measured by Pierce BCA Protein Assay Kit (Thermo Scientific), and 10–20 μg of protein was electrophoresed on 4–15% SDS-PAGE (BIO-RAD). The separated proteins were then transferred onto a PVDF membrane (BIO-RAD), followed by blocking with 5.0% nonfat dry milk in TBST (Tris-buffered saline with Tween 20) (pH 7.4; 0.15 M NaCl, 10 mM Tris-HCl, and 0.1% Tween 20) for 1 h at room temperature. Afterwards, the membrane was incubated overnight at 4 °C with anti-Ca_V_γ4 (1:400; Alomone labs), Ca_V_1.2 (1:500; Sigma), Ca_V_1.3 (1:400; Abcam), Ca_V_α_2_δ1 (1:500; Abcam), Ca_V_β1 (1:500; Abcam), Aldh1a3 (1:1000; Abcam), Cleaved Caspase-3 (1:1000; Cell Signaling), P21 (1:1000; Abcam) antibodies followed by incubation with anti-rabbit IgG (1:2000; Cell Signaling) or goat anti-mouse IgG (1:2000; Dako) at least 1 h at room temperature. Normalization was carried out by incubating membrane with anti-β actin (1:2000; Sigma) or PPIB (1:2000; Abcam) antibodies, or by the corresponding total protein. Immunoreactivity was detected using an enhanced chemiluminescence reaction (Pierce, Rockford, IL, USA) by SuperSignal West Femto Maximum Sensitivity Substrate (Thermo Scientific).

### Immunostaining and confocal imaging

Isolated human islet cells were seeded on glass-bottomed dishes and cultured overnight. Then the cells were fixed by 3% PFA for 30 min and permeablized with Perm Buffer III (BD, USA) for 30 min. The primary antibodies of rabbit anti-Ca_V_γ4 (Abnova), Guinea pig anti-insulin (Eurodiagnostika) were diluted by 1:100 and 1:400, respectively, and incubated with cells overnight at 4 °C. Immunoreactivity was quantified using fluorescently labeled secondary antibodies: Alexa Fluor 647, Donkey anti-rabbit, red (1:300, Jackson ImmunoResearch), Cy 2, Donkey anti-guinea pig, green (1:300, Jackson ImmunoResearch). The Confocal images were acquired using a Zeiss 510 Meta LSM and a ×63 oil immersion objective and the fluorescent intensity was analyzed with software ZEM 2009.

### Chromatin immunoprecipitation

β-TC6 cells were maintained in Dulbecco’s modified Eagle’s medium (DMEM; Invitrogen) supplemented with 10% fetal bovine serum (FBS; Sigma) and 1% penicillin/streptomycin (PEST; Invitrogen). β-TC6 cells were transfected with myc-tagged MAFA and chromatin was prepared 3 days after transfection. Protein/DNA chromatin fragments were immunoprecipitated with rabbit anti-myc antibody (Novus) or mouse IgG (Jackson ImmunoResearch) as previously described^49^. Enrichment was assessed by qPCR (StepOne, Life Technologies) and is presented as percent input. Primer sequences were +1683 bp forward TTTGTGAGGCGTTCTTTCCC, +1683 bp reverse TGTCCTCCAATTCCGAGTCC, −63 bp forward TACAGCCAGTAGTCGGTGC, −63 bp reverse CTATGAGGCGCCCACCAT. Albumin control promoter sequences were not detected in IgG and myc-immunopreciptated DNA.

### Ca^2+^ imaging

Twenty-four hours prior to Ca^2+^ imaging, the cells were transferred to glass-bottom dishes while diluted 1:6 (~1 × 10^5^ cells). Fluo-5F (Kd = 2.3 μM) (Invitrogen) was used for measuring intracellular Ca^2+^ concentration [Ca^2+^]_i_. The cells were loaded at room temperature for 30 min with Fluo-5F (1 μM) dissolved in the perfusion buffer (KRB) supplemented with 5 mM glucose. Stimulation was carried out by 16.7 mM glucose KRB buffer in the absence or presence of DMSO (1:1000) or Bay K8644 (300 nM) or isradipine (2 μM), and/or 70 mM KCl KRB buffer at room temperature. Time lapse region of interest (ROI) images, the mean and peak intensity of ROIs were acquired by confocal microscopy using a ×40 water immersion objective. A ratio was calculated by taking the fluorescence intensity in the time lapse divided by the average fluorescence intensity under pre-stimulatory conditions. The frequency of peak intensity was counted as ratio >1.5, and the time integral of the fluorescence signal (AUC) was calculated by GraphPad software.

### Duolink in situ detection

INS-1 832/13 cells were transferred on the μ-8-well plate (iBidi) 12–24 h before staining experiments. The cells were fixed by 3% PFA for 30 min and permeablized with Perm Buffer III (BD, USA) for 40 min. The primary antibodies of rabbit anti-Ca_V_γ4 (Alomone labs) and mouse anti-Ca_V_1.3 (Abcam) were diluted by 1:100 and 1:200, respectively, and incubated with cells for overnight. The staining protocol followed instructions provided by the manufacturer and the spots were imaged by confocal microscopy and the spots numbers per cell were calculated by Duolink Image Tool (Olink Bioscience, Sweden).

### Measurement of cellular viability (MTT) and apoptosis

The viability of cells was investigated in INS-1 cells silenced with Ca_V_γ4 siRNA for 72 h. Measurement was performed using the MTT reagent kit according to the manufacturer’s instructions (Roche). To measure the cell apoptosis in living cells, 7-AAD (BD Pharmingen) staining was used in 72 h Ca_V_γ4-silenced INS-1 cells, and the positive cells were counted by confocal microscopy to indicate apoptotic cells.

### Statistics

The data are presented as means ± SEM for the indicated number of observations or different experiments, or presented as box-plots with whiskers created using Tukey's method. The significance of random differences was analyzed by Student’s *t*-test. Pearson correlation coefficient (*R*) was used and tested (*t*-test) for a measure of the linear correlation between expression of genes. Holm–Bonferroni correction was used to adjust the rejection criteria of each of the individual comparisons for multiple groups, and one-way ANOVA with Tukey’s test was used for multiple comparisons. *p* < 0.05 was considered as significant. *p < 0.05; **p < 0.01; ***p < 0.001.

### Reporting Summary

Further information on experimental design is available in the Nature Research Reporting Summary linked to this article.

## Supplementary information

Supplementary information

Description of Supplementary Data 1

Supplementary Data 1

Reporting Summary

## Data Availability

All human islet microarray data are MIAME compliant, and the raw data have been deposited in the Gene Expression Omnibus (GEO) database, www.ncbi.nlm.nih.gov/geo (accession no. GSE50398, GSE38642, and GSE44035). All source data underlying the graphs presented in the main figures are available as Supplementary Data. All other data generated and/or analyzed during the current study are available from the corresponding author on reasonable request.
