# Supplementary material for: The calcium channel subunit gamma-4 is regulated by MafA and necessary for pancreatic beta-cell specification
Source: Commun Biol. 2019 Mar 15;2:106. doi: 10.1038/s42003-019-0351-4 (PMC6420573; doi:10.1038/s42003-019-0351-4)
Supplement: Supplementary file 1 — Supplementary information [file 42003_2019_351_MOESM1_ESM.pdf]

## Supplementary Figure 1

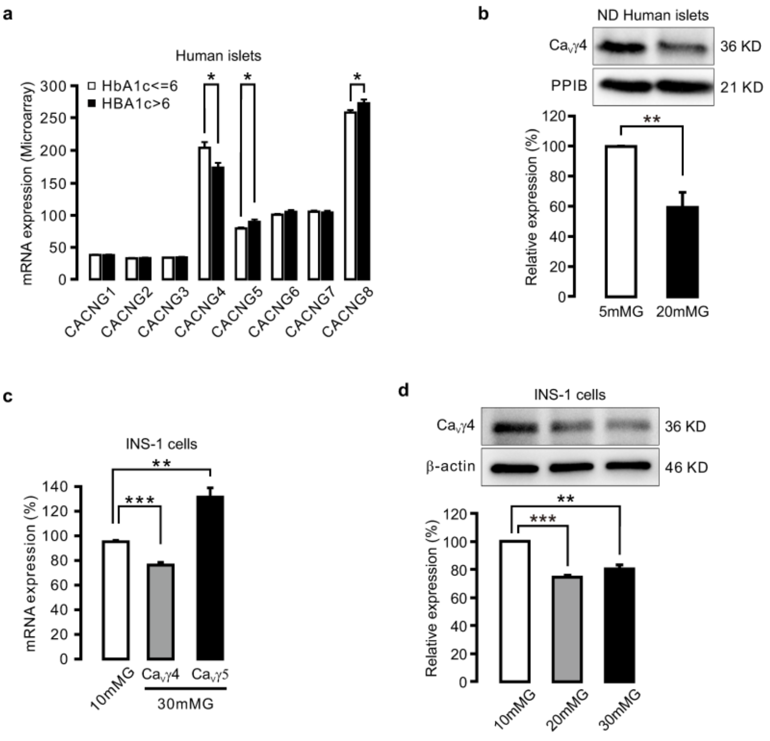


**Supplementary Figure 1. Caᵥγ4 expression is reduced in beta-cells in response to glucotoxicity.** **(a)** Microarray gene profile showing all Caᵥγ subunit genes mRNA expression in human pancreatic islets. n = 77 donors for HbA1c ≤ 6, 36 donors for HbA1c > 6, *p = 0.028 (Caᵥγ4), *p = 0.012 (Caᵥγ5), *p = 0.037 (Caᵥγ8). **(b)** Decreased Caᵥγ4 protein expression in ND human islets cultured at 5 or 20 mM glucose (48h). n = 3 donors, **p = 0.003. See also Supplementary figure 7l, but cultured for 72h. n = 5 donors, not significant. **(c)** Caᵥγ4 and Caᵥγ5 mRNA expression in INS-1 cells cultured at 10 or 30 mM glucose for 24h. n = 3, ***p < 0.001 (Caᵥγ4), **p = 0.001 (Caᵥγ5). **(d)** Immunoblotting of Caᵥγ4 in INS-1 cells cultured at 10, 20 or 30 mM glucose for 72 h. n = 4, ***p < 0.001 (20 mMG), **p = 0.009 (30 mMG). Data are presented as Mean ± SEM and were analyzed with two-tailed unpaired Student's *t*-test; and the significance in (c-d) were corrected by Holm-Bonferroni method.

## Supplementary Figure 2

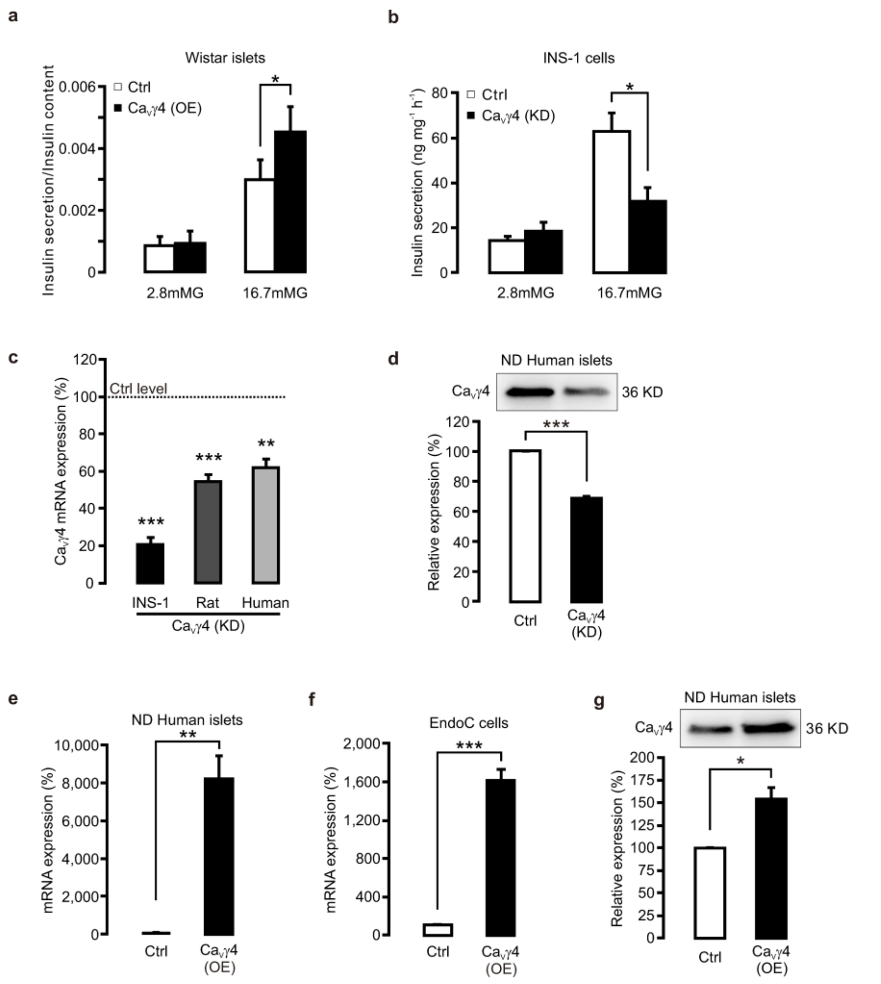


**Supplementary Figure 2. Successful siRNA and lentiviral transfection of Ca$_V$γ4 in human beta-cells and the effects on glucose-stimulated insulin secretion (GSIS). (a)** GSIS in Ca$_V$γ4 overexpressed Wistar rat islets. n = 3, *p = 0.027. **(b)** As in (a) but in Ca$_V$γ4 silenced INS-1 cells. n = 4, *p = 0.024. **(c)** Ca$_V$γ4 mRNA expression in Ca$_V$γ4 silenced INS-1 cells, Wistar rat islets and ND human islets to validate the silencing effect of Ca$_V$γ4 siRNA. n = 7 (INS-1, ***p < 0.001), 4 (rat, ***p < 0.001) and 3 (human, **p = 0.001). **(d)** Ca$_V$γ4 protein expression in Ca$_V$γ4 silenced ND human islets normalized by total protein (see Supplementary figure 7n). n = 3 donors, ***p < 0.001. **(e)** Ca$_V$γ4 mRNA expression in Ca$_V$γ4 overexpressed ND human islets to validate the Ca$_V$γ4 lentiviral overexpressing efficiency. n = 3 donors, **p = 0.002. **(f)** As in (e) but in Ca$_V$γ4 overexpressed EndoC cells. n = 4, ***p < 0.001. **(g)** As in (d) but in Ca$_V$γ4 overexpressed ND human islets normalized by total protein (see Supplementary figure 7o). n = 3 donors, *p = 0.014. Data are presented as Mean ± SEM and were analyzed with two-tailed paired (a) and unpaired (b-g) Student's *t*-test.

**Supplementary Figure 3**

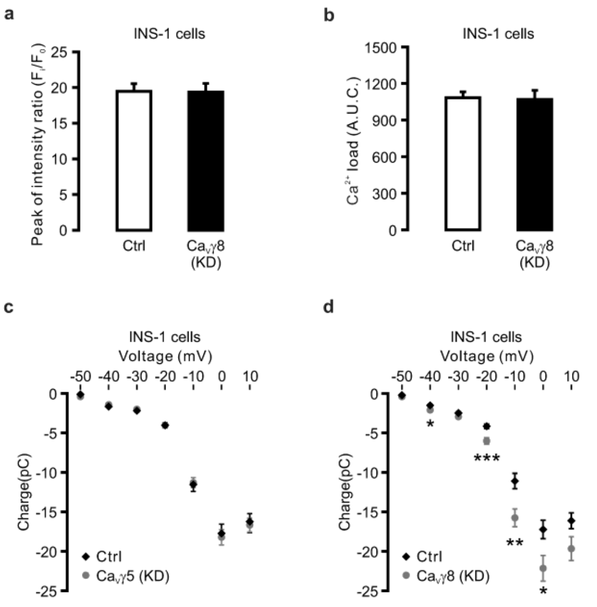


**Supplementary Figure 3. $Ca^{2+}$ influx in Cavγ8 silenced INS-1 cells. (a)** Comparison of intracellular $Ca^{2+}$ concentration $[Ca^{2+}]_i$ peak intensity (Fi/F0) in control and Cavγ8 silenced INS-1 cells by $Ca^{2+}$ imaging, $p = 0.957$. **(b)** Integrated $Ca^{2+}$ load (Area Under the Curve, A.U.C.) after the stimulation of 70 mM KCl, 0-180s, $p = 0.867$, see Figure 3f. $n = 62$ control and 42 Cavγ8 silencing cells, from 3 independent experiments, for both (a) and (b). **(c)** Integrated whole-cell $Ca^{2+}$ charge-voltage relations in Cavγ5 silenced INS-1 cells. $n = 53$ control and 57 Cavγ5 silencing cells from 3 independent experiments. **(d)** As in (c) but in Cavγ8 silenced INS-1 cells. $n = 41$ control and 40 Cavγ8 silencing cells from 3 independent experiments, $^*p = 0.031$ (-40mV), $^{***}p < 0.001$ (-20mV), $^{**}p = 0.003$ (-10mV), $^*p = 0.017$ (0mV). Data are presented as Mean ± SEM and were analyzed with two-tailed unpaired Student's *t*-test.

## Supplementary Figure 4

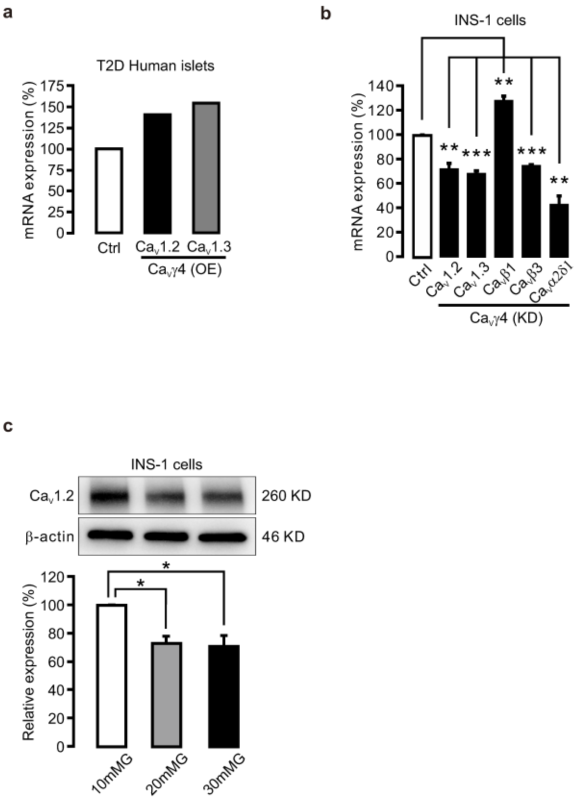


**Supplementary Figure 4. $Ca_V\gamma4$ regulates $Ca_V1.2$ and $Ca_V1.3$ expression. (a)** Increased tendency of $Ca_V1.2$ and $Ca_V1.3$ mRNA expression in $Ca_V\gamma4$ overexpressed T2D human islets, n = 1 donor. **(b)** $Ca_V$ channels mRNA expression in $Ca_V\gamma4$ silenced INS-1 cells. n = 3, $^{**}p = 0.004$ ($Ca_V1.2$), $^{***}p < 0.001$ ($Ca_V1.3$), $^{**}p = 0.002$ ($Ca_V\beta1$), $^{***}p < 0.001$ ($Ca_V\beta3$), $^{**}p = 0.001$ ($Ca_V\alpha_2\delta1$). **(c)** $Ca_V1.2$ protein expression in INS-1 cells cultured with 10, 20 or 30 mM glucose. n = 4, $^{*}p = 0.012$ (20 mMG), $^{*}p = 0.028$ (30 mMG). Data are presented as Mean ± SEM and were analyzed with two-tailed unpaired Student's *t*-test; and the significance in (a-b) were corrected by Holm-Bonferroni method.

## Supplementary Figure 5

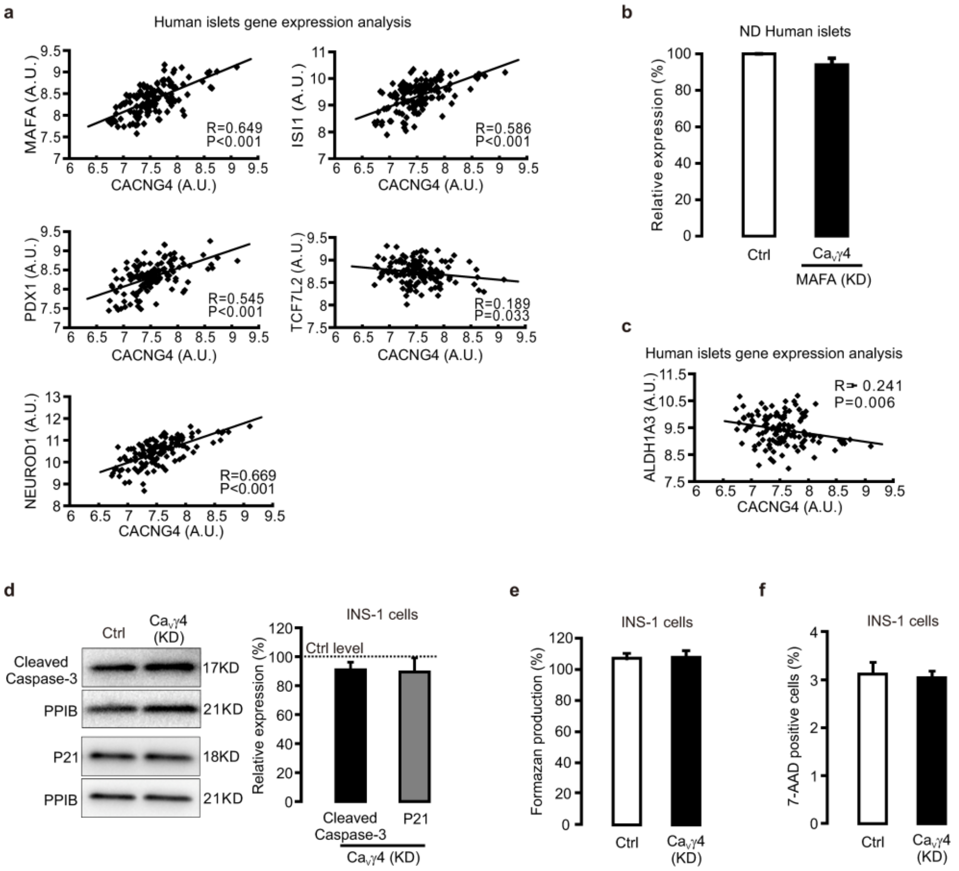


**Supplementary Figure 5. Effect of Ca$_V\gamma$4 on beta-cell health.** **(a)** Correlation of mRNA expressions (Microarray) between Ca$_V\gamma$4 (*CACNG4*) and transcription factors *MAFA*, *ISL1*, *PDX1*, *TCF7L2* and *NEUROD1* in human islets. n = 128 donors. Pearson correlation coefficient (*R*) was tested (*t*-test) and labeled alongside with p values. **(b)** Ca$_V\gamma$4 mRNA expression in MAFA silenced ND human islets. n = 3 donors, p = 0.237. **(c)** Correlation of mRNA expressions (Microarray) between Ca$_V\gamma$4 (*CACNG4*) and *ALDH1A3* in human islets. n = 128 donors. **(d)** Cleaved Caspase-3 and P21 immunoblotting and means of expression in Ca$_V\gamma$4 silenced INS-1 cells. n = 4 each, p = 0.155 and p = 0.356, respectively. **(e)** Cellular viability (formazan production) measured in Ca$_V\gamma$4 silenced INS-1 cells. n = 4, p = 0.945. **(f)** Cell viability test by 7-AAD staining in Ca$_V\gamma$4 silenced INS-1 cells. n = 3, p = 0.782. Data are presented as Mean ± SEM and were analyzed with two-tailed unpaired Student's *t*-test.

## Supplementary Figure 6

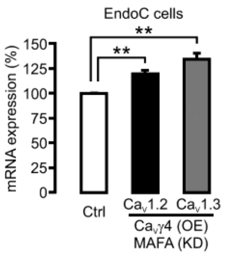


**Supplementary Figure 6. Ca$_V$1.2 and Ca$_V$1.3 expression in Ca$_V\gamma$4 overexpressed and MAFA silenced beta-cells.** Increased Ca$_V$1.2 and Ca$_V$1.3 mRNA expression in Ca$_V\gamma$4 overexpressed and simultaneously MAFA silenced human EndoC cells. n = 4, **p = 0.002 (Ca$_V$1.2), **p = 0.001 (Ca$_V$1.3). Data are presented as Mean ± SEM and were analyzed with two-tailed unpaired Student's *t*-test; and the significance were corrected by Holm-Bonferroni method.

# Supplementary Figure 7

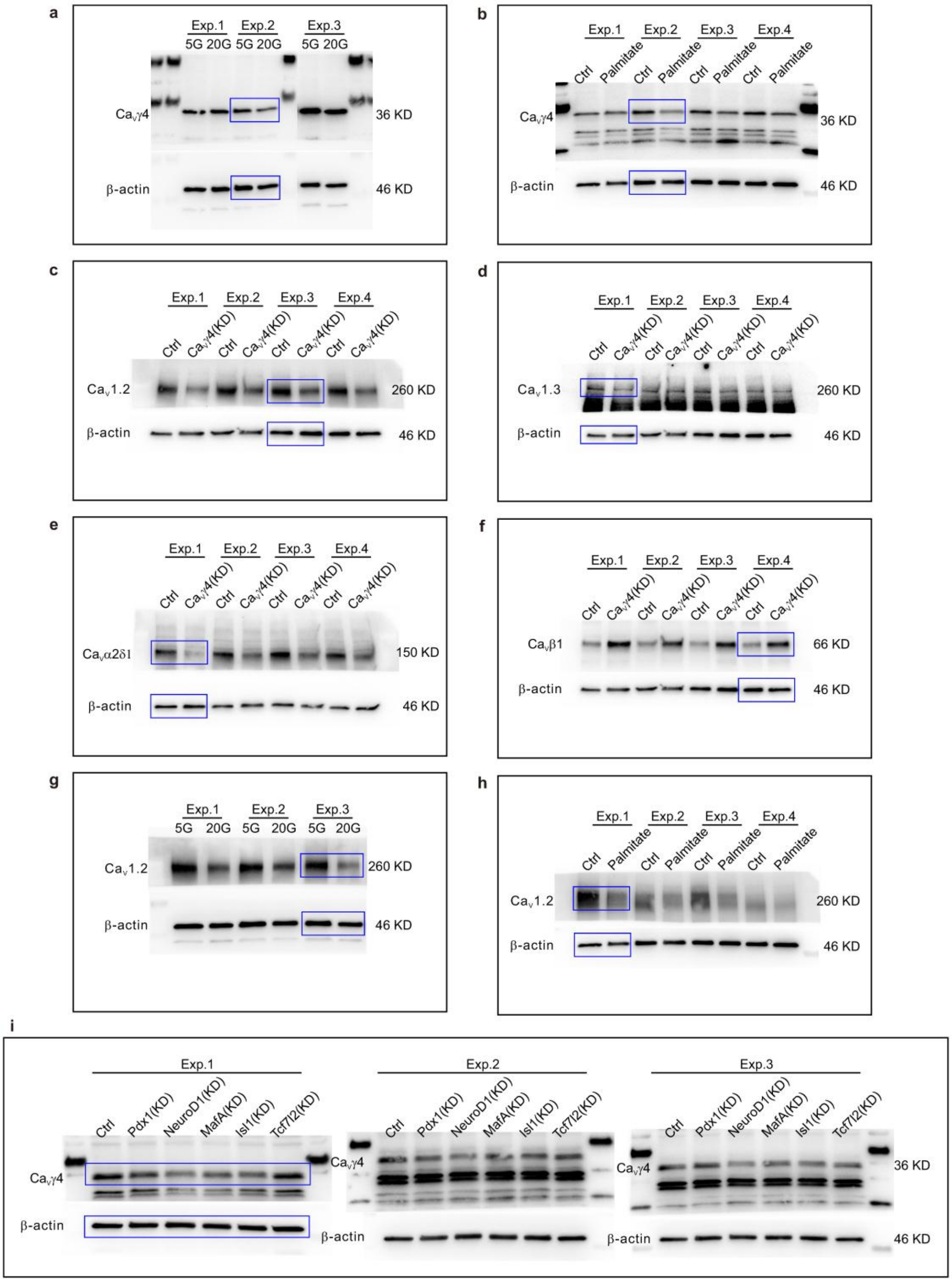

## Supplementary Figure 7

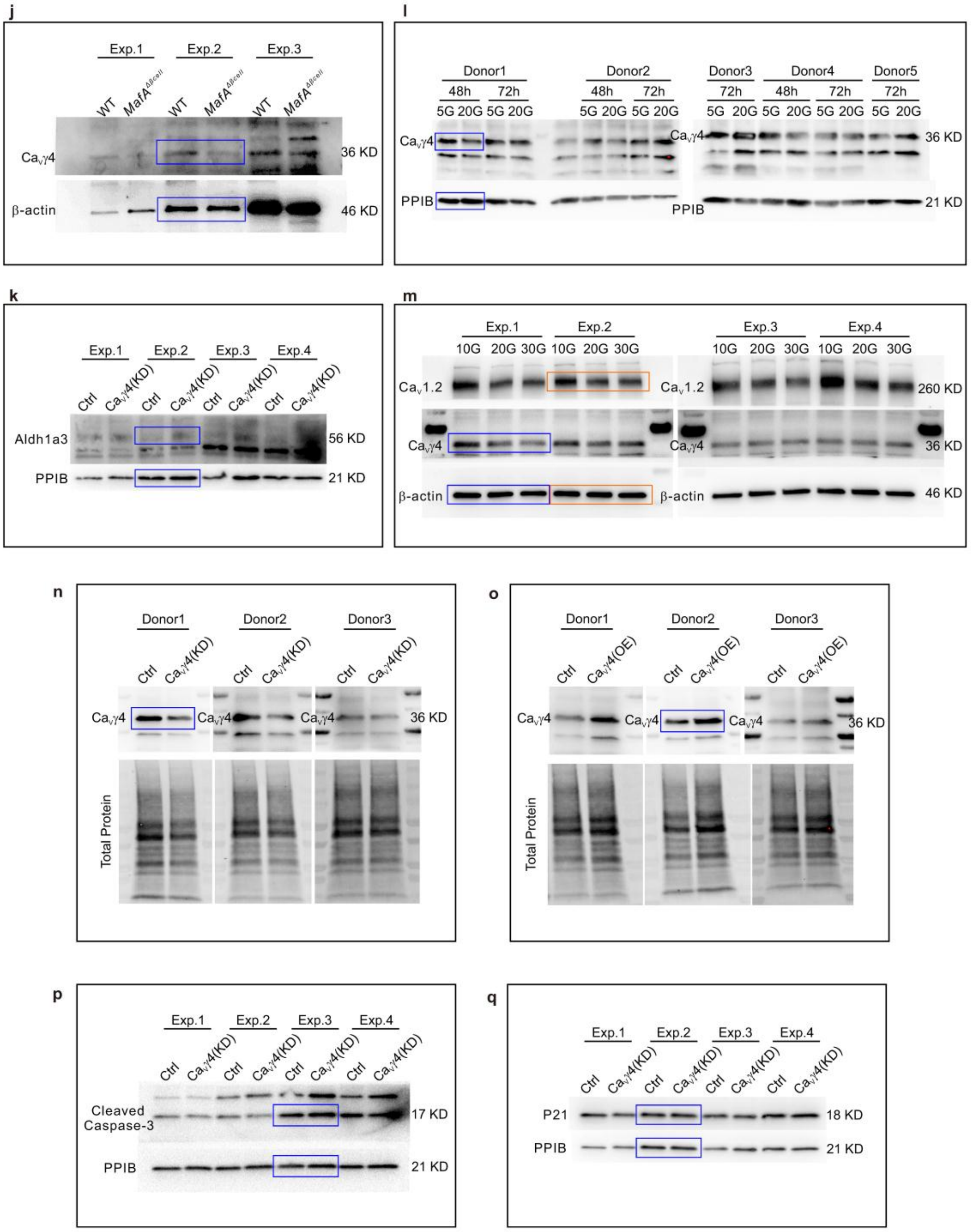


**Supplementary Figure 7. Full pictures of protein blots presented in the main paper.** Protein blots from which the blue or orange boxes correspond, with similar band order, to Figure 1g-h **(a-b)**, Figure 4d **(c-f)**, Figure 4e-f **(g-h)**, Figure 5c-d and h **(i-k)**, Supplementary Figure 1b and d **(l and m (blue))**, Supplementary Figure 2d and g **(n-o)**, Supplementary Figure 4c **(m (orange))**, and Supplementary Figure 5d **(p-q)**.

**Supplementary Table 1**

Supplementary Table 1. Characteristics of human islet donors used for experiment.

| | All (n=51) | Non-diabetic (n=37) | T2D (n=14) |
|---|---|---|---|
| Age (years) | 61 ± 11 | 60 ± 12 | 64 ± 9 |
| Sex (female/male) | 20/31 | 16/21 | 4/10 |
| BMI (kg $m^{-2}$) | 27.1 ± 5.7 * | 26.1 ± 4.7 * | 29.5 ± 7.4 |
| HbA1c (%) | 5.9 ± 0.8 ** | 5.5 ± 0.4 ** | 6.9 ± 0.5 |
| Purity (%) | 73 ± 20 | 70 ± 21 | 78 ± 12 |
| Days cultured | 3 ± 1 | 3 ± 1 | 2 ± 1 |

Data are presented as mean ± s.d.

* 1 missing value

** 2 missing values
