# Supplementary material for: The calcium channel subunit gamma-4 is regulated by MafA and necessary for pancreatic beta-cell specification
Source: Commun Biol. 2019 Mar 15;2:106. doi: 10.1038/s42003-019-0351-4 (PMC6420573; doi:10.1038/s42003-019-0351-4)
Supplement: Supplementary file 2 — Description of Supplementary Data 1 [file 42003_2019_351_MOESM2_ESM.pdf]

Description of Supplementary Data

File name: Supplementary Data 1

Description: The Supplementary Data 1 file contains all source data underlying the graphs and charts presented in the main figures (Figure 1-6). The source data includes: Relative mRNA expressions, Fluorescence intensity, Relative protein expressions, Insulin secretion, The increase of beta-cell membrane capacitance, The whole cell Ca^{2+} currents, Intracellular Ca^{2+} concentration ($[Ca^{2+}]_i$) peak intensity, Integrated Ca^{2+} load, Frequency of $[Ca^{2+}]_i$ peaks, Human microarray mRNA expressions and Correlation of microarray mRNA expressions.
