# Supplementary material for: The calcium channel subunit gamma-4 is regulated by MafA and necessary for pancreatic beta-cell specification
Source: Commun Biol. 2019 Mar 15;2:106. doi: 10.1038/s42003-019-0351-4 (PMC6420573; doi:10.1038/s42003-019-0351-4)
Supplement: Supplementary file 4 — Reporting Summary [file 42003_2019_351_MOESM4_ESM.pdf]

nature research

Corresponding author(s): Erik Renström

Last updated by author(s): Feb 5, 2019

# Reporting Summary

Nature Research wishes to improve the reproducibility of the work that we publish. This form provides structure for consistency and transparency in reporting. For further information on Nature Research policies, see Authors & Referees and the Editorial Policy Checklist.

## Statistics

For all statistical analyses, confirm that the following items are present in the figure legend, table legend, main text, or Methods section.

| n/a | Confirmed | |
| --- | --- | --- |
| ☐ | ☒ | The exact sample size (*n*) for each experimental group/condition, given as a discrete number and unit of measurement |
| ☐ | ☒ | A statement on whether measurements were taken from distinct samples or whether the same sample was measured repeatedly |
| ☐ | ☒ | The statistical test(s) used AND whether they are one- or two-sided<br>*Only common tests should be described solely by name; describe more complex techniques in the Methods section.* |
| ☒ | ☐ | A description of all covariates tested |
| ☐ | ☒ | A description of any assumptions or corrections, such as tests of normality and adjustment for multiple comparisons |
| ☐ | ☒ | A full description of the statistical parameters including central tendency (e.g. means) or other basic estimates (e.g. regression coefficient) AND variation (e.g. standard deviation) or associated estimates of uncertainty (e.g. confidence intervals) |
| ☐ | ☒ | For null hypothesis testing, the test statistic (e.g. *F*, *t*, *r*) with confidence intervals, effect sizes, degrees of freedom and *P* value noted<br>*Give P values as exact values whenever suitable.* |
| ☒ | ☐ | For Bayesian analysis, information on the choice of priors and Markov chain Monte Carlo settings |
| ☒ | ☐ | For hierarchical and complex designs, identification of the appropriate level for tests and full reporting of outcomes |
| ☐ | ☒ | Estimates of effect sizes (e.g. Cohen's *d*, Pearson's *r*), indicating how they were calculated |

*Our web collection on statistics for biologists contains articles on many of the points above.*

## Software and code

Policy information about availability of computer code

| | |
| --- | --- |
| Data collection | Zeiss ZEN 2009 (Carl Zeiss Microscopy, Germany. For Confocal images acquisition), Pulse + X-Chart Extension (version 8.6 or later; HEKA, Lambrecht-Pfalz, Germany. For recording cell electrophysiological activities), QuantStudio™ Software V1.3 (Applied Biosystems, USA. For qPCR measurement), Megellan 7.1 SP1 (Tecan Life Sciences, Switzerland. For Absorbance detection of ELISA). |
| Data analysis | Zeiss ZEN 2009 (Carl Zeiss Microscopy, Germany. For fluorescence intensity measurement), Pulse + X-Chart Extension (version 8.6 or later; HEKA, Lambrecht-Pfalz, Germany. For capacitance measurement), Microsoft Excel 2016, GraphPad Prism 7 and SPSS 22.0 (For data calculation and statistics) |

For manuscripts utilizing custom algorithms or software that are central to the research but not yet described in published literature, software must be made available to editors/reviewers. We strongly encourage code deposition in a community repository (e.g. GitHub). See the Nature Research guidelines for submitting code & software for further information.

## Data

Policy information about availability of data

All manuscripts must include a data availability statement. This statement should provide the following information, where applicable:

- Accession codes, unique identifiers, or web links for publicly available datasets
- A list of figures that have associated raw data
- A description of any restrictions on data availability

All human islet microarray data are MIAME compliant, and the raw data have been deposited in the Gene Expression Omnibus (GEO) database, www.ncbi.nlm.nih.gov/geo (accession no. GSE50398, GSE38642 and GSE44035). All source data underlying the graphs presented in the main figures are available as Supplementary Data. All other data generated and/or analyzed during the current study are available from the corresponding author on reasonable request.

# Field-specific reporting

Please select the one below that is the best fit for your research. If you are not sure, read the appropriate sections before making your selection.

☒ Life sciences ☐ Behavioural & social sciences ☐ Ecological, evolutionary & environmental sciences

For a reference copy of the document with all sections, see nature.com/documents/nr-reporting-summary-flat.pdf

# Life sciences study design

All studies must disclose on these points even when the disclosure is negative.

| | |
|---|---|
| Sample size | The sample size used in current study was calculated using PS-Power and Sample size Calculation softwares. Please see the Figure Legends and Materials & Methods (Statistics). |
| Data exclusions | No samples were excluded from this study but observations detected as significant outliers using GraphPad online QuickCalcs software. The analysis was performed on the observed values. |
| Replication | All measurements were performed on at least 3 times by different batches of human donor islets, rat or mouse islets and different culture passages of INS-1 832/13 cells; except the results of insulin secretion, calcium currents and qPCR in Cacng4 overexpressed T2D human islets (from 1 donor). Because fresh T2D human islets acquisition is very difficult, but at least 3 technical repeats were conducted. All measurements on samples from in vitro experiments were performed with at least 2 technical replicates. |
| Randomization | For the in vivo experiments MafA knockout mice were age and sex matched with C57/Black WT controls. The same as GK and Wistar rats, db/db and C57/Black mice, Akita and WT mice. |
| Blinding | The in vivo experiments (islets isolation) were performed by an experienced laboratory technician who gave the samples to a PhD student. The student then conducted the experiments later on using the samples in a blinded manner. |

# Reporting for specific materials, systems and methods

We require information from authors about some types of materials, experimental systems and methods used in many studies. Here, indicate whether each material, system or method listed is relevant to your study. If you are not sure if a list item applies to your research, read the appropriate section before selecting a response.

## Materials & experimental systems

| n/a | Involved in the study | |
|---|---|---|
| ☐ | ☒ | Antibodies |
| ☐ | ☒ | Eukaryotic cell lines |
| ☒ | ☐ | Palaeontology |
| ☐ | ☒ | Animals and other organisms |
| ☒ | ☐ | Human research participants |
| ☒ | ☐ | Clinical data |

## Methods

| n/a | Involved in the study | |
|---|---|---|
| ☒ | ☐ | ChIP-seq |
| ☒ | ☐ | Flow cytometry |
| ☒ | ☐ | MRI-based neuroimaging |

## Antibodies

| | |
|---|---|
| Antibodies used | Anti-Cacng4 (#ACC-114, Alomone labs and #PAB9886, Abnova), Anti-Cacna1c (#C1603, Sigma), Anti-Cacna1d (#ab85491, Abcam), Anti-Cacna2d1 (#ab2864, Abcam), Anti-Cacnb1 (#ab85020, Abcam), Anti-Aldh1a3 (#ab129815, Abcam), Anti-Cleaved Caspase-3 (#9661, Cell Signaling), Anti-P21 (#ab109199, Abcam), Anti-β actin (#A5441, Sigma), Anti-PPIB (#ab16045, Abcam) and Anti-insulin (Eurodiagnostika) antibodies. |
| Validation | All antibodies used in this study were commercially available antibodies with documented selectivity. Detailed validation please see the manufactuer's website (sources listed above and in the Methods). |

## Eukaryotic cell lines

Policy information about cell lines

| | |
|---|---|
| Cell line source(s) | The rat insulinoma INS-1 832/13 cells were used and were originally donated by Dr. C. B. Newgaard, Duke University, USA. Please see Method part for details. |
| Authentication | INS-1 cell line is the most widely used and studied clonal beta-cell line with over 2700 citations in PUBMed, and of which 168 for subclone 832/13. |

| | |
|---|---|
| Mycoplasma contamination | Mycoplasma test is performed in Lund University routinely. |
| Commonly misidentified lines (See ICLAC register) | This is not relevant to us. |

## Animals and other organisms

Policy information about studies involving animals; ARRIVE guidelines recommended for reporting animal research

| | |
|---|---|
| Laboratory animals | 1. The MafA β-cell conditional knockout mice was described previously (Ganic, E. et al. Cell reports. 2016).<br>2. Wistar and GK male rats (Charles River Laboratories, Wilmington, MA) 6-11 week of age was used.<br>3. Adult db/db and control (C57/bl) mice (Janvier Laboratory, France), and Akita (Ins2+/-) and wild type (Ins2+/+) male littermates (Jackson laboratories, stock number 003548) were used (7-13 weeks) for the indicated experiment. |
| Wild animals | The study did not involve wild animals. |
| Field-collected samples | The study did not involve samples collected from the field. |
| Ethics oversight | All procedures were approved by the ethics committees at Uppsala and Lund Universities in Sweden. |

Note that full information on the approval of the study protocol must also be provided in the manuscript.
